# PCNA–RECQL5–RPRD1B recruit repair components to stressed replication forks to promote survival of BRCA1-deficient cancer cells

**DOI:** 10.1093/nar/gkag831

**Published:** 2026-08-27

**Authors:** Manh Tien Tran, Elizabeth A Williamson, Aruna S Jaiswal, Dominic Arris, Nhat Nguyen, Andrew Carrillo, Shaun Olsen, Kimi Kong, Montasser Shaheen, Doraid Sadadeen, Daohong Zhou, Yaxia Yuan, Shahrez Syed, Youngho Kwon, Sameer Salunkhe, Patrick Sung, Robert Hromas

**Affiliations:** Department of Medicine and the Mays Cancer Center, the University of Texas Health Science Center San Antonio, San Antonio, TX 78229, United States; Department of Medicine and the Mays Cancer Center, the University of Texas Health Science Center San Antonio, San Antonio, TX 78229, United States; Department of Medicine and the Mays Cancer Center, the University of Texas Health Science Center San Antonio, San Antonio, TX 78229, United States; Department of Medicine and the Mays Cancer Center, the University of Texas Health Science Center San Antonio, San Antonio, TX 78229, United States; Department of Medicine and the Mays Cancer Center, the University of Texas Health Science Center San Antonio, San Antonio, TX 78229, United States; Department of Medicine and the Mays Cancer Center, the University of Texas Health Science Center San Antonio, San Antonio, TX 78229, United States; Department of Biochemistry and Structural Biology, the Center for Innovative Drug Discovery, and the Greehey Children’s Cancer Research Institute, the University of Texas Health Science Center San Antonio, San Antonio, TX 78229, United States; Department of Medicine and the Mays Cancer Center, the University of Texas Health Science Center San Antonio, San Antonio, TX 78229, United States; Department of Medicine and the Mays Cancer Center, the University of Texas Health Science Center San Antonio, San Antonio, TX 78229, United States; Department of Medicine and the Mays Cancer Center, the University of Texas Health Science Center San Antonio, San Antonio, TX 78229, United States; Department of Biochemistry and Structural Biology, the Center for Innovative Drug Discovery, and the Greehey Children’s Cancer Research Institute, the University of Texas Health Science Center San Antonio, San Antonio, TX 78229, United States; Department of Biochemistry and Structural Biology, the Center for Innovative Drug Discovery, and the Greehey Children’s Cancer Research Institute, the University of Texas Health Science Center San Antonio, San Antonio, TX 78229, United States; Department of Biochemistry and Structural Biology, the Center for Innovative Drug Discovery, and the Greehey Children’s Cancer Research Institute, the University of Texas Health Science Center San Antonio, San Antonio, TX 78229, United States; Department of Biochemistry and Structural Biology, the Center for Innovative Drug Discovery, and the Greehey Children’s Cancer Research Institute, the University of Texas Health Science Center San Antonio, San Antonio, TX 78229, United States; Department of Biochemistry and Structural Biology, the Center for Innovative Drug Discovery, and the Greehey Children’s Cancer Research Institute, the University of Texas Health Science Center San Antonio, San Antonio, TX 78229, United States; Department of Biochemistry and Structural Biology, the Center for Innovative Drug Discovery, and the Greehey Children’s Cancer Research Institute, the University of Texas Health Science Center San Antonio, San Antonio, TX 78229, United States; Department of Medicine and the Mays Cancer Center, the University of Texas Health Science Center San Antonio, San Antonio, TX 78229, United States

## Abstract

Given their defect in homologous recombination, BRCA1-deficient cancers use polymerase theta (POLQ) microhomology-mediated end joining (MMEJ) for repair of stressed replication forks. We found that BRCA1-deficient cells repress microRNA (miR) 4485-3p, and reconstituting expression of this miR resulted in the death of BRCA1-deficient cells. In our interrogation of the mechanism of this synthetic lethality, we discovered that miR-4485-3p suppresses expression of RPRD1B protein. Depletion of RPRD1B led to selective BRCA1-deficient cell death, decreased MMEJ, and decreased replication fork repair after replication stress. RPRD1B promotes the recruitment of the MMEJ components 53BP1, PARP1, and POLQ to the stressed replication fork. However, RPRD1B does not have a canonical DNA-binding domain, and our search for the mechanism of its recruitment to stressed replication forks discovered that this is mediated by RECQL5 and ubiquitinated (Ub) PCNA. Thus, our results unveil an essential signaling cascade at the stressed replication fork in BRCA1-deficient cancer cells, in which Ub PCNA recruits RECQL5, which recruits RPRD1B to mediate the assembly of the MMEJ repair apparatus. These data suggest that this repair scaffold may be targeted in the development of a novel synthetic therapy of BRCA1-deficient cancers.

## Graphical Abstract

**Graphical Abstract:**
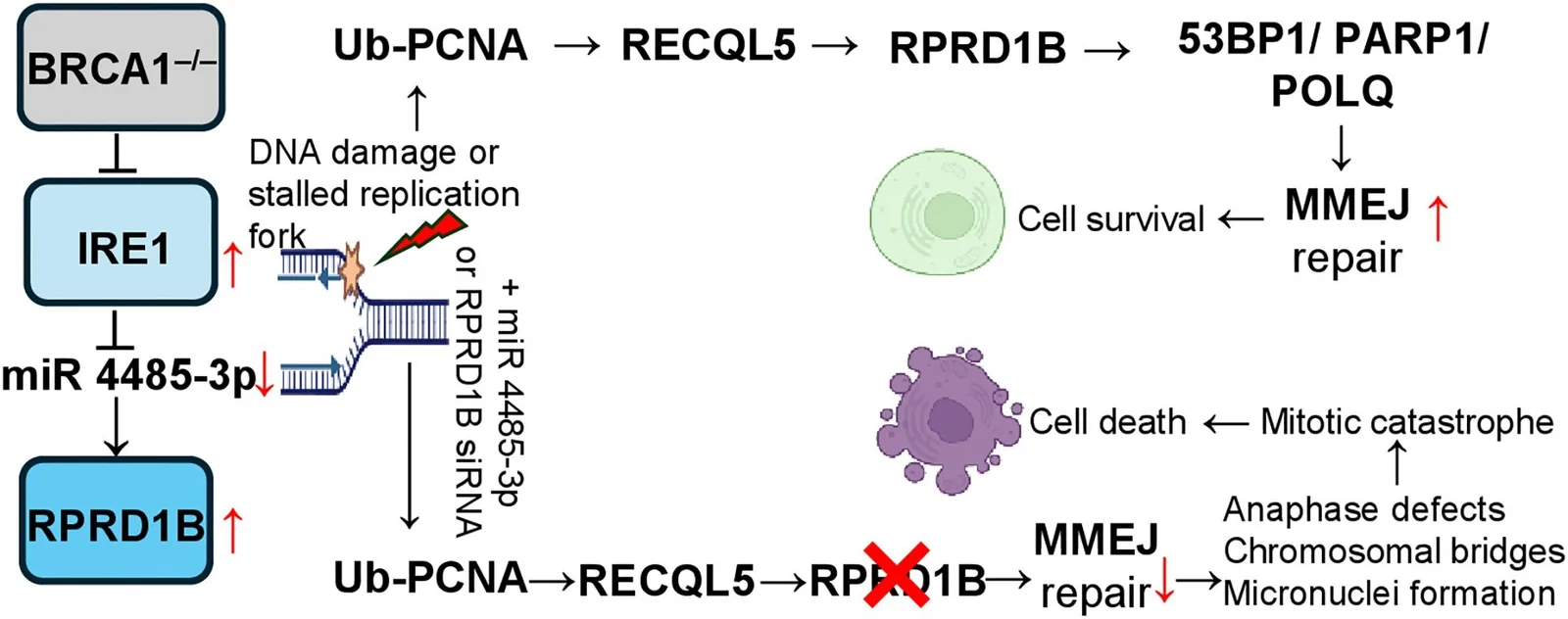

## Introduction

BRCA1 is a key factor in homologous recombination (HR) repair of DNA double-strand breaks (DSBs) in cycling cells [1]. Inherited and somatic mutations in BRCA1 cause genomic instability and lead to breast or ovarian cancers [2]. BRCA1 has pleiotropic activities in maintaining genome stability [3]. The BRCT phosphoprotein-binding domain of BRCA1 localizes it to regions of DNA DSBs, and the BRCA1-BARD1 complex possesses an E3 ubiquitin ligase activity that promotes the assembly of HR components at DSBs. BRCA1-CtIP promotes 5′ end resection at DSBs to promote 3′ strand invasion for HR, whereas the 53BP1-associated protein complex opposes this end resection and thereby enhances non-homologous end joining (NHEJ) repair [3].

HR is the preferred pathway for stressed replication fork repair and restart because it is conservative, and this is crippled in cells deficient in BRCA1 [3, 4]. BRCA1-deficient (def) cells can become genomically unstable and ultimately undergo neoplastic transformation because they have lost conservative DSB repair and have increased replication stress from many sources, including unresolved R-loops, impairment of conservative stressed replication fork repair and restart, and increased stalled fork degradation [5]. Therefore, BRCA1-def cells require other mechanisms to repair and restart stressed replication forks for their survival. BRCA1-def cells can survive and progress toward neoplastic transformation by relying on error-prone repair pathways, such as microhomology-mediated end joining (MMEJ), to resolve stressed replication forks [6–8].

MMEJ, which substantially overlaps with alternative NHEJ, initiates when PARP1 outcompetes the Ku complex for free DNA ends of a DSB and promotes short 5′ end resection by the MRN complex [9]. POLQ mediates alignment of microhomologies of ∼2–20 nt, and non-homologous tails are cleaved by FEN1 or ERRC1/XPF [6–10]. Gaps are filled in by POLQ, and then strands are ligated by LIG3/XRCC1 [11]. MMEJ is invariably error-prone due to the short homology required for annealing and cleavage of the unannealed overhanging ends, often leading to deletions or less frequent insertions [12, 13]. We and others found that this pathway also mediates most chromosomal translocations [14]. MMEJ can be recruited for stressed replication fork repair and restart when there is overwhelming damage, or when HR is compromised, such as in BRCA1-def cells [6–8, 12, 15]. Consistent with this dependency, several components of the MMEJ/fork repair pathway, including FEN1, have been implicated as therapeutic vulnerabilities in BRCA-def tumors, supporting the concept that disruption of end processing and repair can be synthetically lethal in this context [16–18].

We previously discovered that the microRNase IRE1 was a target of the E3 ligase activity of BRCA1-BARD1 for ubiquitination and subsequent degradation [19]. In BRCA1-def cells, this led to the overexpression and auto-activation of the IRE1 RNase, which digested a cohort of microRNAs (miRs) in these cells [20, 21]. We reconstituted the expression of these repressed miRs in BRCA1-def cells and found three, miR-223-3p, miR-4638-5p, and miR 4485–3p, that induce synthetic lethality in BRCA1-def but not wild-type (WT) BRCA1-proficient (pro) daughter cells. MiR-223-3p was lethal to BRCA1-def cells from repression of MMEJ by decreasing translation of PSO4, PARP1, and CtIP [20]. MiR-4638-5p was lethal to BRCA1-def cells by repressing translation of the structure-specific RNase, TATDN2, resulting in impaired resolution of R-loops and increased replication stress [21]. These studies imply that IRE1 activation permits BRCA1-def cells to survive the loss of HR by repressing miRNAs that affect the efficiency of alternate repair pathways that such cells require to repair damaged DNA and replication forks [20, 21]. In this study, we report that RPRD1B is a target of miR-4485-3p and that it proves to be essential for the survival of BRCA1-def cancer cells.

RPRD1B (also termed CREPT, and a homolog of yeast rtt103) was initially identified via its interaction with the C-terminal domain of RNA polymerase II and regulation of that domain’s acetylation and phosphorylation [22]. RPRD1B is preferentially expressed in many tumor types over normal tissues, where it can serve as a prognostic factor [23]. Several groups have reported that RPRD1B promotes NHEJ repair [24, 25]. Previous proteomic analysis using tandem affinity purification and mass spectroscopy found that RPRD1B interacts with multiple DNA repair components, including the Ku complex, DNA-PKcs, PARP1, MSH2, RAD23B, SMC1A, SSBP1, DDB1, and PCNA [26]. The loss of RPRD1B also decreases MSH2 stability and results in impaired mismatched repair [26]. Thus, although RPRD1B does not have a canonical DNA-binding domain, it interacts with many DNA repair pathway components, which suggests that it could function as a general scaffold for DNA damage repair machineries.

In this study, we found that miR-4485-3p represses the translation of RPRD1B mRNA, and that loss of RPRD1B leads to the abrogation of MMEJ DSB repair, decreased stressed replication fork repair and restart, increased mitotic catastophe, and death of BRCA1-def but not BRCA1-WT-proficient cells. We discovered that RECQL5 recruits RPRD1B to the stressed replication fork, where it initiates assembly of the MMEJ apparatus.

## Materials and methods

### Full methods are available in the Supplement

#### MiRNA, siRNA, plasmids, and transfection

All reagents are listed in the Supplement. Transfection took place as described [19–21].

#### Luciferase assay for assessing RPRD1B protein stability

Luciferase assay for protein stability was performed as described [20].

#### Colony formation assay

Clonogenic assays were performed as previously described [19–21, 27, 28].

#### Ubiquitinated PCNA, His-RPRD1B, and RECQL5 purification

Recombinant tagged RPRD1B, PCNA, and RECQL5 proteins were prepared as described [29].

#### Protein extraction and western blot analysis

Western analysis was performed as described [19–21]. All antibody sources are defined in the Supplement.

#### Co-immunoprecipitation and chromatin isolation assays

Co-immunoprecipitation (Co-IP) experiments were performed as described [19–21, 30]. Chromatin isolation experiments were performed as described [28]. All antibodies and reagents are defined in the Supplement.

#### Immunofluorescence, nuclear structure assays, and DNA damage foci

Confocal immunofluorescence of nuclear structures and DNA damage was performed as described [19–21, 27, 28]. All antibodies and reagents are defined in the Supplement. For nuclear structure assays and DNA damage foci assays, confocal analysis of DAPI-stained cells was used to access nuclear abnormalities, including micronuclei and nuclear bridging, as previously described [27, 28]. All reagents are listed in the Supplement.

#### I-SceI-based MMEJ reporter assay: EJ2-GFP

U2OS EJ2-GFP cells were used to measure the repair of an I-SceI-induced DSB using MMEJ as described [31].

#### Isolation of proteins on nascent DNA

Isolation of proteins on nascent DNA (IPOND) was performed as described previously [21, 27, 28]. All reagents are listed in the Supplement.

#### DNA fiber assays

DNA fiber analysis was performed to assess replication fork arrest and restart as described [21, 27, 28]. All reagents are listed in the Supplement.

#### *In vitro* protein pulldown assays

FLAG-RECQL5 or HA-RPRD1B was immunoprecipitated from MDA-MB-436 cells as described [21, 30]. After incubation together *in vitro*, each of the purified proteins were immunoprecipitated in turn and analyzed by western analysis for the partner as described [21, 30]. All antibodies are listed in the Supplement.

### Statistical analyses

ImageJ was used for densitometric detection of western immunoblot signals compared to loading controls for statistical analysis [32]. All data are represented as mean ± standard deviation (SD) from at least three independent experiments, unless otherwise specified. For all statistically significant comparisons, *P* values are displayed in the figures. Statistical significance is indicated in the figures using asterisks (*P ≤ .05; **P ≤ .01; ***P ≤ .001). Unless otherwise indicated, a two-sided Student’s *t*-test was used for statistical analysis of each experiment. All experiments were performed independently at least three times.

## Results

### Mir-4485-3p reconstitution in BRCA1-deficient cells induces synthetic lethality

**Figure 1.**
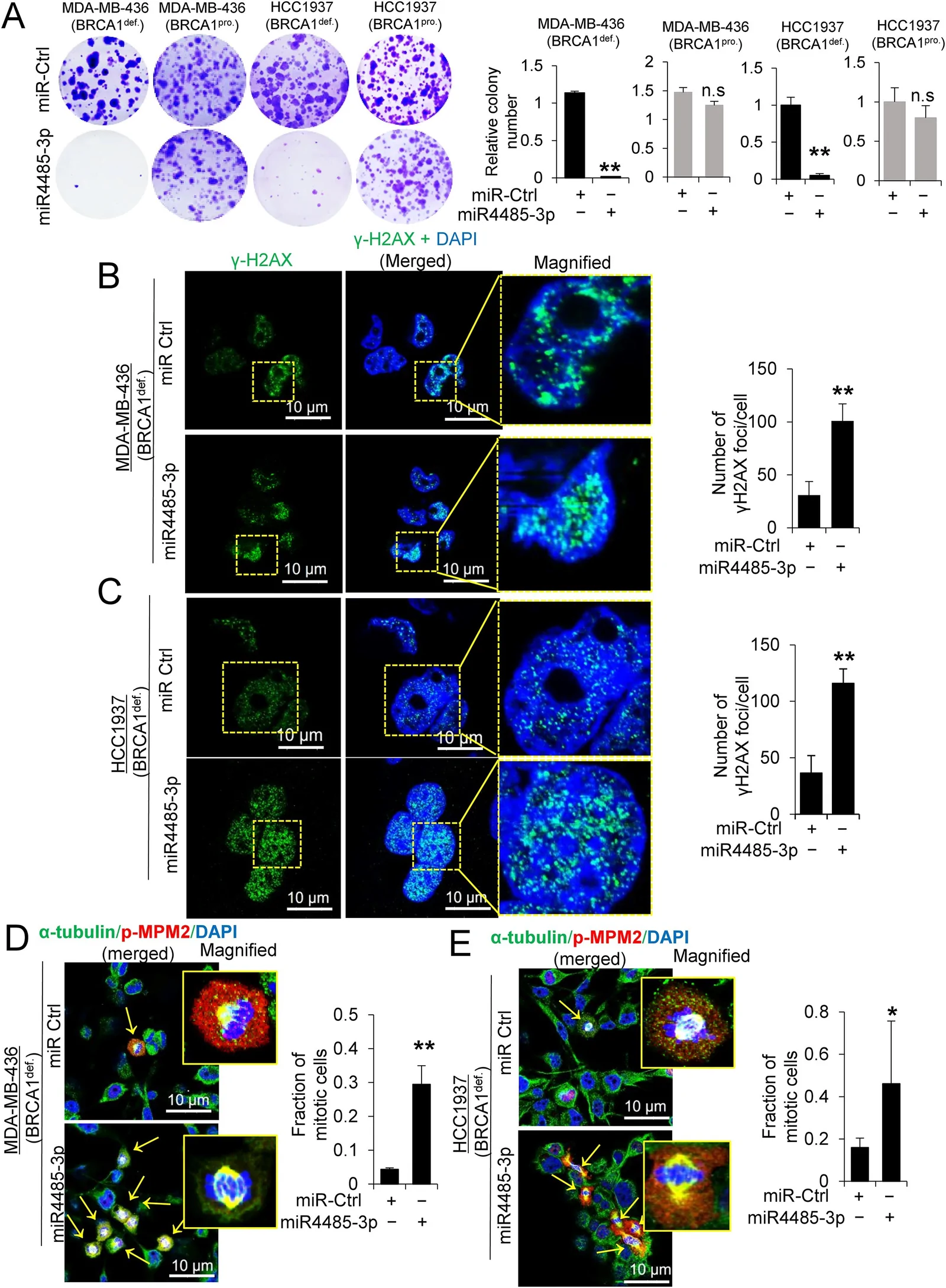
Reconstitution of miR4485-3p induces metaphase arrest and decreases cell survival in BRCA1-deficient breast cancer cells. (**A**) Representative images (left) and quantification (right) of clonogenic survival assays in BRCA1-deficient (def) and BRCA1-reconstituted (pro) MDA-MB-436 and HCC1937 cells following transfection with either 50 nM miR-Ctrl or miR-4485-3p. Data represent the mean ± SD from three independent experiments performed in triplicate, unless otherwise indicated. **P ≤ .01; n.s. = not significant. Analysis of γ-H2AX foci formation in BRCA1-def MDA-MB-436 cells (**B**) and BRCA1-def HCC1937 cells (**C**) following transfection with either 50 nM miR-Ctrl or miR-4485-3p. Cells were stained for γ-H2AX (green) and DAPI (blue). At least 100 nuclei were analyzed per condition. Data shown are representative of five independent experiments (*n* = 5). **P ≤ .01. Scale bar, 10 µm. Representative confocal immunofluorescence images and quantification of mitotic arrest, predominantly at anaphase, in BRCA1-def MDA-MB-436 (**D**) and HCC1937 (**E**) cells following transfection with either 50 nM miR-Ctrl or miR-4485-3p. Cells were stained for α-tubulin (green), p-MPM2 (red), and DAPI (blue). Arrows indicate arrested cells, most in anaphase. At least 100 cells per condition were analyzed per condition. The representative data were obtained from five independent experiments (*n* = 5). *P ≤ .05, **P ≤ .01. Scale bar, 10 µm.

We previously found that the microRNase IRE1 is overexpressed in BRCA1-deficient (def) cancer cells [19] and depleting it increased the expression of multiple miRNAs [20, 21], including miR-4485-3p (Supplementary Fig. S1A). Reconstituting miR-4485-3p induces lethality in the BRCA1-def MDA-MB-436 and HCC1937 cancer cell lines but not in their daughter cell line counterparts that have had WT BRCA1 genetically repleted, which we refer to as BRCA1-proficient (pro) (Fig. 1A) [21]. Since BRCA1-def cancer cells have lost HR and thus are already deficient in DSB repair, any further loss of DNA repair capacity can be harmful [1, 3, 5]. Therefore, we asked whether increased miR-4485-3p expression would lead to BRCA1-def cell death via interference with residual DSB repair capacity as measured by γ-H2AX foci. We found that reconstituting miR-4485-3p in the BRCA1-def versus their BRCA1-pro daughter cell lines indeed resulted in a marked increase γ-H2AX foci (3.2-fold increase in Fig. 1B and 3.2-fold increase in Fig. 1C), as determined by confocal immunofluorescent histology. In contrast, WT BRCA1-pro daughter cells did not show a significant increase in γ-H2AX foci (no increase in Supplementary Fig. S1B and 1.3-fold increase in Supplementary Fig. S1C) when miR4485-3p was overexpressed.

Since unresolved DSBs induce chromosomal abnormalities and subsequent mitotic abnormalities, especially in HR-def cells [33], we next tested whether this increase in DNA DSBs by reconstitution of miR-4485-3p in MDA-MB-436 or HCC1937 BRCA1-def cells would result in failure to complete mitosis using confocal immunofluorescent histology. We found that significantly more BRCA1-def cells became arrested in anaphase (7.6-fold increase in Fig. 1D and 3.1-fold increase in Fig. 1E) after reconstitution of miR-4485-3p than scrambled siRNA (Small interfering RNA) control cells, implying that increased DNA damage was sufficient to slow chromosomal segregation. In contrast, the BRCA1-pro daughter cells did not show a significant increase in mitotic arrest (1.2-fold increase in Supplementary Fig. S1D and 1.3-fold increase in Supplementary Fig. S1E) when miR4485-3p was overexpressed. Consistent with these findings, cell cycle analyses following miR-4485-3p reconstitution showed that BRCA1-def cells underwent G2/M arrest, whereas BRCA1-pro cells and BRCA1 WT MCF7 cells did not exhibit significant cycle changes (Supplementary Fig. S1F). Thus, BRCA1-def cells cannot tolerate increases in miR-4485-3p levels and survive, consistent with its degradation by the higher IRE1 levels in BRCA1-def cells.

**Figure 2.**
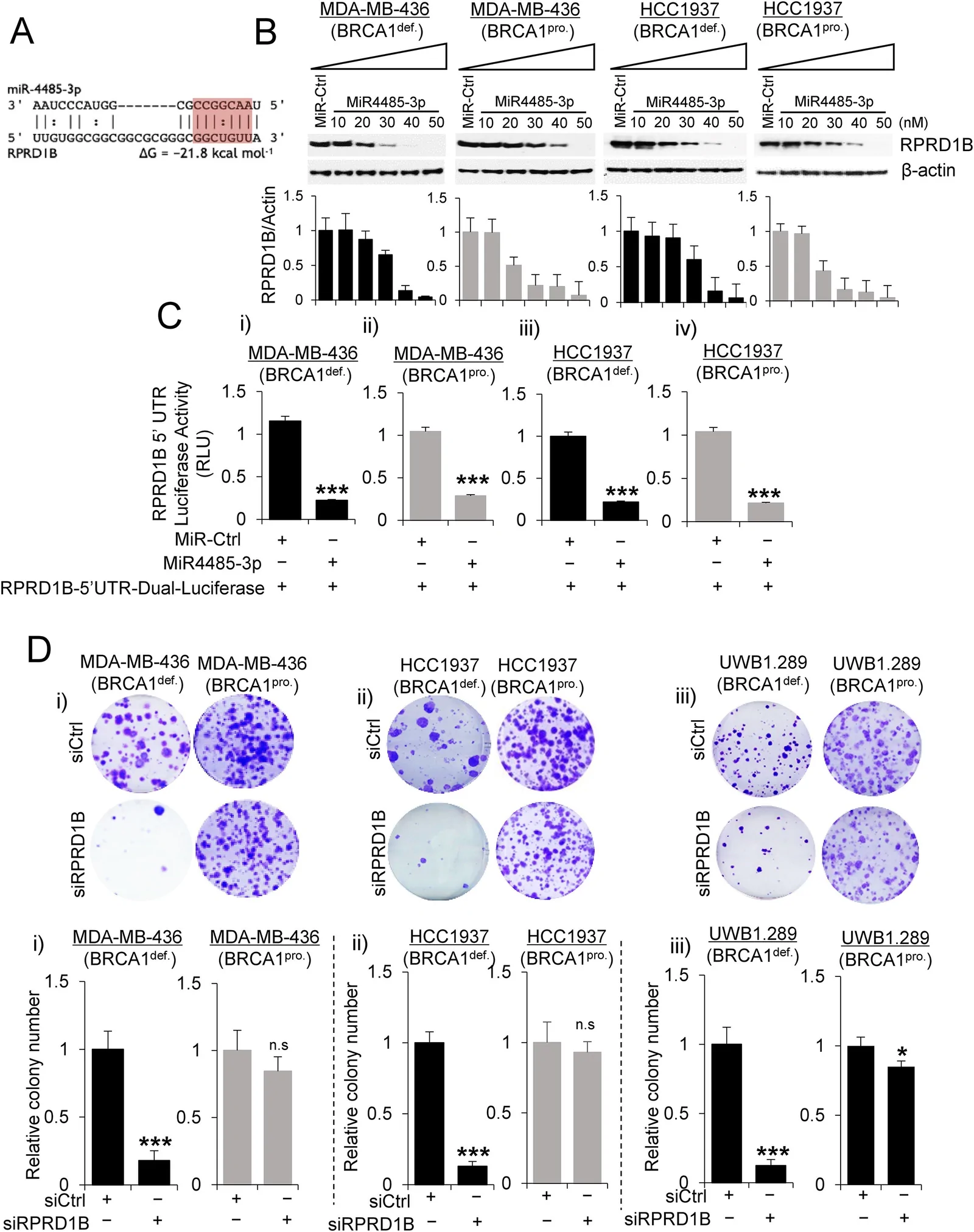
miR-4485-3p represses RPRD1B, whose depletion results in synthetic lethality in BRCA1-def cells. (**A**) Predicted pairing of miR-4485-3p to the 5′ UTR of *RPRD1B* mRNA. (**B**) Western blot (WB) analyses showing repression of RPRD1B protein expression following transfection with miR-4485-3 in miR-4485-3p in BRCA1-def and BRCA1-pro MDA-MB-436 and HCC1937 cells. Densitometric quantification of RPRD1B expression (bottom) normalized to β-actin as a loading control is shown as mean ± SD from three independent experiments. (**C**) miR-4485-3p represses translation via the 5′ UTR of *RPRD1B* mRNA. Luciferase reporter assay of the *RPRD1B* 5′ UTR following transfection with a miR-4485-3p mimic in (i) BRCA1-def MDA-MB-436, (ii) BRCA1-pro MDA-MB-436, (iii) BRCA1-def HCC1937, and (iv) BRCA1-pro HCC1937 cells; *n* = 3 independent transfections, ***P ≤ .001. (**D**) Representative images (top) and quantification (bottom) of clonogenic survival assays in BRCA1-def and BRCA1-pro MDA-MB-436, HCC1937, and UWB1.289 cells following transfection with either siCtrl or siRPRD1B, *n* = 3 independent experiments performed in triplicate, *P ≤ .05, **P ≤ .01, ***P ≤ .001, n.s. = not significant.

We virtually searched for the target of miR-4485-3p whose expression would be required for the survival of BRCA1-def but not BRCA1-pro cancer cells. We discovered that miR-4485-3p was predicted to bind to the 5′ untranslated region (UTR) of RPRD1B at mRNA nucleotides 15–41 from NM_021215.4 with a reasonable energy delta, −21.8 kcal/mol (Fig. 2A). RPRD1B is an RNA Polymerase 2 (RNAP2) C-terminal binding protein that has been reported to promote NHEJ and mismatched repair pathways by undefined mechanisms [22–25]. Since RPRD1B was known to play a role in several DNA repair pathways, we surmised that its downregulation by miR-4485-3p was responsible for the synthetic lethality seen in BRCA1-def cancer cells (Fig. 1A). Reconstituting expression of miR-4485-3p in both the MDA-MB-436 or HCC1937 BRCA1-def cancer cells and their BRCA1-pro daughter cells resulted in a concentration-dependent decrease in RPRD1B protein levels (Fig. 2B), but the expression levels of the related RPRD1A protein remained unaffected (Supplementary Fig. S2A). Transfecting miR-4485-3p into the above BRCA1-def and BRCA1-pro cell lines with a luciferase reporter under the control of the RPRD1B 5′ UTR resulted in decreased luciferase expression, demonstrating that the predicted RPRD1B binding sequence is germane for translational repression by miR-4485-3p in different cell types (Fig. 2C).

### Depletion of RPRD1B causes replication stress and death in BRCA1-def cells

We next explored whether RPRD1B had a role in the survival of BRCA1-def cancer cells and could be a mechanism of cell death from the reconstitution of miR-4485-3p. We found that depletion of RPRD1B in three BRCA1-def cell lines, MDA-MB-436, HCC1937, and UWB1.289, was also highly lethal, but there was significantly less cell death when RPRD1B was depleted in these cell lines with reconstituted WT BRCA1 (Fig. 2D). This demonstrated that only BRCA1-def cells require appropriate levels of RPRD1B to survive [19–21]. To further confirm the specificity of this phenotype, we performed rescue experiments by overexpressing HA-RPRD1B following RPRD1B knockdown in BRCA1-def cells. We found that RPRD1B overexpression rescued cell survival as measured by colony formation assay (Supplementary Fig. S2B). In addition, we used a second independent siRNA (Small interfering RNA) targeting RPRD1B (siRPRD1B #2) and observed a similar reduction in colony formation, further supporting the specificity and reproducibility of the phenotype (Supplementary Fig. S2C).

We also demonstrated that, similar to miR-4485-3p reconstitution in BRCA1-def cells, depletion of RPRD1B led to increased DSBs as measured by γ-H2AX foci in the three BRCA1-def cell lines (2.3-fold increase in Supplementary Fig. S2D, 3.6-fold increase in Supplementary Fig. S2E, and 2.7-fold increase in Supplementary Fig. S2F). However, there was little change in γ-H2AX foci in the BRCA1-pro counterpart cells upon RPRD1B depletion (1.3-fold increase in Supplementary Fig. S3A, 1.3-fold increase in Supplementary Fig. S3B, and 1.3-fold increase in Supplementary Fig. S3C).

**Figure 3.**
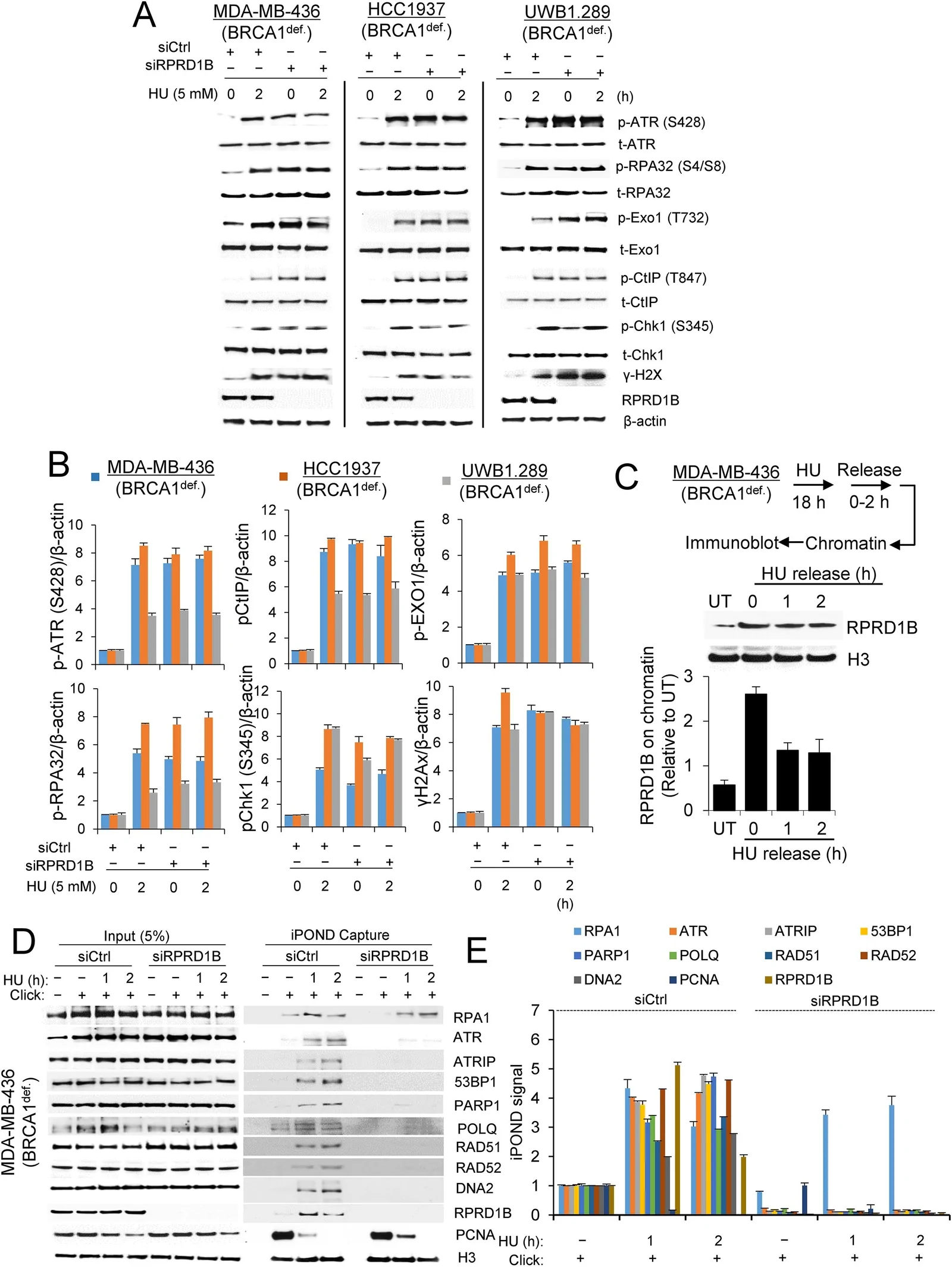
RPRD1B depletion results in replication stress in BRCA1-def cells. (**A, B**) RPRD1B depletion activates biomarkers of replication stress in BRCA1-def MDA-MB-436, HCC1937, and UWB1.289 cells. WB analysis of phosphorylated ATR (S428), RPA32 (S4/S8), Exo1 (T732), CtIP (T847), Chk1 (S345), and γ-H2AX in cells treated with hydroxyurea (HU) or mock-treated, with or without RPRD1B depletion. Representative immunoblots are shown in panel (A), and densitometric quantification normalized to β-actin from three independent experiments is presented in panel (B) as means ± SD (**C**) RPRD1B localizes to chromatin upon replication stress. Chromatin fractions were isolated from BRCA1-def MDA-MB-436 cells at 0, 1, and 2 h following HU release and analyzed for RPRD1B levels by WB. Histone H3 served as the chromatin loading control. Densitometric analysis represents mean ± SD from three independent experiments, with RPRD1B levels normalized to H3. (**D, E**) iPOND analysis of RPRD1B localization at replication forks in BRCA1-def MDA-MB-436 cells. Cells were transfected with either (30 nM) siCtrl or (30 nM) siRPRD1B for 2 days and subsequently subjected to iPOND assay. Replicating DNA was pulse-labeled for 20 min, followed by treatment with 5 mM HU for the indicated times (0, 1, or 2 h) to induce replication fork stalling. Proteins associated with newly synthesized DNA were isolated and analyzed by WB for RPA1, ATR, ATRIP, 53BP1, PARP1, POLQ, RAD51, RAD52, RPRD1B, γ-H2AX, PCNA, and Histone H3, in which γ-H2AX and PCNA served as markers of replication stress. Histone H3 served as a loading control. (E) Densitometric analysis of iPOND-associated RPRD1B signals from three independent experiments is presented as mean ± SD.

Since depletion of RPRD1B resulted in synthetic lethality in BRCA1-def cancer cells, and the most well-defined pathway for synthetic lethality in these cells is with the PARP1 inhibitors generating intolerable replication stress [34], we asked whether RPRD1B depletion causes replication stress. We measured phosphorylation of multiple components of stressed replication fork signaling and repair by WB analysis (Fig. 3A and B). We found that loss of RPRD1B in BRCA1-def cancer cells resulted in the constitutive phosphorylation of ATR, RPA32, EXO1, CtIP, CDK1, and γ-H2AX even without a replication stressor present. Given that these biomarkers of replication stress became constitutively activated, we concluded that RPRD1B is necessary for preventing general replication stress in BRCA1-def cancer cells. Adding the replication stressor (HU) did not appreciably increase the phosphorylation of these replication fork repair components, indicating that loss of RPRD1B alone leads to high levels of replication stress in the BRCA1-def cells (Fig. 3A and B).

RPRD1B depletion in BRCA1-pro cell lines also resulted in the constitutive phosphorylation of CtIP, RPA32, γ-H2AX, CDK1, and CHK1, but not ATR or EXO1 (Supplementary Fig. S3D and E). We also examined 53BP1, ATM, and CHK2 phosphorylation under the same conditions and did not observe significant changes (Supplementary Fig. S3F and G), suggesting that RPRD1B depletion preferentially activates replication stress-associated signaling rather than ATM/CHK2-mediated and 53BP1-mediated DSB responses in BRCA1-def cells.

In contrast, RPRD1B depletion in BRCA1 WT MCF7 cells did not result in appreciable changes in the phosphorylation level of ATR, RPA32, CtIP, CDK1, γ-H2AX, ATM, or CHK2 under the same conditions (Supplementary Fig. S3H and I), suggesting that BRCA1-WT cells do not require RPRD1B to repair stressed replication forks. Consistently, colony formation assay showed no significant difference between siCtrl- and siRPRD1B-treated MCF7 cells (Supplementary Fig. S3J), indicating that RPRD1B depletion does not impair long-term proliferative capacity of BRCA1-WT cells.

We next assessed whether RPRD1B is recruited to chromatin after replication stress with HU treatment of cells. We found that RPRD1B is recruited to chromatin upon replication stress in BRCA1-def cells (Fig. 3C) and in BRCA1-WT HEK293 cells (Supplementary Fig. S4A and B), further indication that RPRD1B may function in genomic repair in cells that are either BRCA-def or BRCA1-pro, but it is not as important for cell survival in the latter case. Next, we used iPOND to assess whether RPRD1B is recruited to stressed replication forks [19, 27, 28]. This analysis showed that RPRD1B is present at stressed replication forks in both BRCA1-def and BRCA1-pro cells (Supplementary Fig. S4C–F), and this correlates with the presence of γ-H2AX at forks. We then asked whether RPRD1B was important for recruitment of components of stressed replication fork repair in BRCA1-def cancer cells using iPOND (Fig. 3D and E). Interestingly, we found that when RPRD1B was depleted, even though RPA and PCNA were still present at stressed replication forks, ATRIP, ATR, 53BP1, PARP1, POLQ, RAD51, RAD52, and DNA2 were noticeably absent. In addition, iPOND analyses showed that XRCC1 and LIG3 recruitment to stressed replication forks was also markedly reduced following RPRD1B depletion in BRCA1-def cells (Supplementary Fig. S4G and H), further supporting a role for RPRD1B in organizing replication fork-associated repair complexes in alternative end-joining/MMEJ repair. Together, these findings suggest that RPRD1B recruitment is an essential and possibly sufficient early step in the assembly of stressed replication fork repair machinery in BRCA1-def cells.

Next, we performed iPOND analysis in BRCA1-WT MCF7 cells following RPRD1B depletion. In contrast to BRCA1-def cells, RPRD1B depletion did not appreciably alter the abundance of RPA1, ATR, 53BP1, PARP1, or POLQ at replication forks (Supplementary Fig. S4I), consistent with the lack of replication stress signaling and DNA damage phenotypes observed in these cells.

### RPRD1B is required for stressed replication fork repair and restart in BRCA1-deficient cells

**Figure 4.**
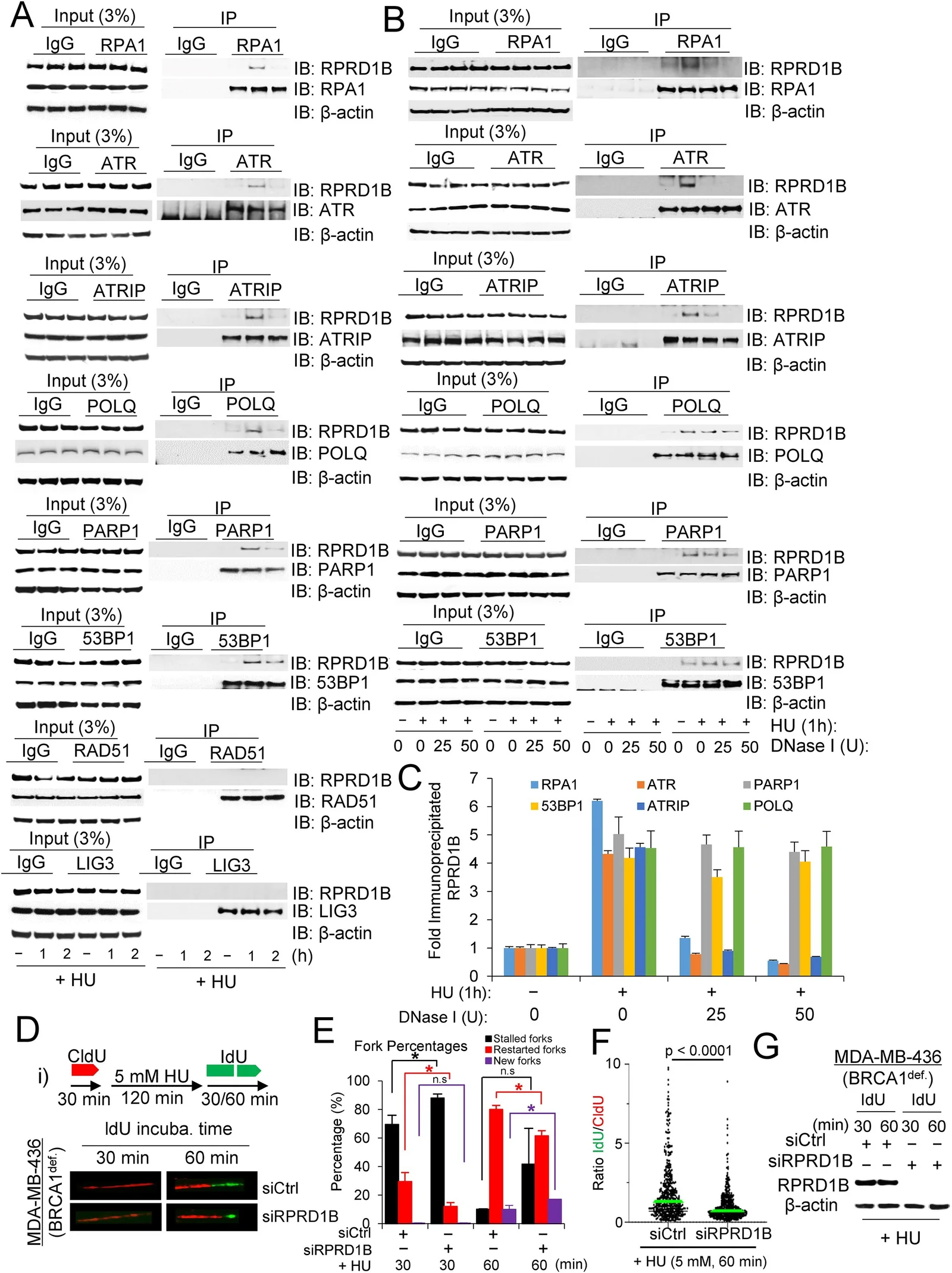
RPRD1B interacts with replication fork repair components and promotes replication fork repair and restart in BRCA1-def cells. (**A**) RPRD1B co-immunoprecipitates with replication fork repair components upon replication stress. BRCA1-def MDA-MB-436 cells treated with 5 mM HU for the indicated times (0, 1, or 2 h) were subjected to Co-IP analysis. Antibodies against RPA1, ATR, ATRIP, POLQ, PARP1, 53BP1, RAD51, DNA Ligase III (LIG3), or control IgG were used to pull down proteins. WB analyses were subsequently performed using the corresponding antibodies to detect specific proteins. Whole-cell lysates (WCLs; total protein extracts from cells prior to immunoprecipitation) were used, with ~3% loaded as input control. (**B**) RPRD1B interactions with replication fork repair components are resistant to DNase I treatment. BRCA1-def MDA-MB-436 cells were treated with HU for 1 h, followed by treatment of cell lysates with DNase I (0, 25, or 50 U) for 30 min at 37°C prior to Co-IP analysis. Antibodies against RPA1, ATR, ATRIP, POLQ, PARP1, 53BP1, or control IgG were used to pull down proteins, followed by WB analysis of the indicated repair protein components. WCLs were used, with ~3% loaded as input control. (**C**) Densitometric quantification of Co-IP signals corresponding to panel (B), showing RPRD1B interactions with RPA1, ATR, ATRIP, POLQ, PARP1, or 53BP1. Data represent mean ± SD from three independent experiments. (**D, E**) Experimental schema and representative images of DNA fiber assays from siCtrl- and si-RPRD1B-transfected BRCA1-def MDA-MB-436 cells. Cells were pulsed-labeled with CldU for 30 min (red), treated with 5 mM HU for 120 min, and subsequently pulse-labeled with IdU (green) for 30 and 60 min. Analysis of stalled, restarted, and newly initiated replication forks by DNA fiber assay (*n* = 3). Data are represented as mean ± SD from three independent experiments, *P ≤ .05, ***P ≤ .001, n.s. = not significant. (**F**) Analysis of DNA fiber length ratios (IdU/CldU), following 60 min of IdU labeling in siCtrl-or siRPRD1B-transfected BRCA1-def MDA-MB-436 cells from three independent experiments. RPRD1B-depleted cells exhibited shorter restarted tracts, indicating impaired replication fork restart and/or decreased fork progression following restart. (**G**) WB analysis showing RPRD1B protein depletion in BRCA1-def MDA-MB-436 cells corresponding to data shown in panels (D–F).

The decreased recruitment of multiple stressed replication fork repair components when RPRD1B is depleted implies that it serves as a scaffold upon which the replication fork repair apparatus is assembled. This would be consistent with its previously reported utility in multiple DNA repair pathways [24–26]. Therefore, we asked whether RPRD1B interacts with these replication fork repair components *in vivo*. We found that RPRD1B coimmunoprecipitates with ATR, ATRIP, 53BP1, POLQ, and PARP1, but not LIG3 or RAD51 during replication stress induced by HU treatment in BRCA1-def cells (Fig. 4A and Supplementary Fig. S5A). A heterodimeric partner of RPRD1B, RPRD1A, also interacted with RPA1 but not PARP1, POLQ, or 53BP1 (Supplementary Fig. S5B and C).

However, we found that the interaction of ATR, ATRIP, and RPA with RPRD1B did not survive treatment of the immunoprecipitate with DNase1, indicating that replication fork DNA may mediate their interaction with RPRD1B, as could occur if these components were positioned in proximity on DNA. Interestingly, our data showed RPRD1B interactions with PARP1, 53BP1, and POLQ remained intact after treatment with DNase1 (Fig. 4B and C), demonstrating that these interactions could occur independently of DNA bridging and likely reflect more stable protein–protein associations within the replication stress response complex. We noted that the interaction of these repair components with RPRD1B decreased at 2 h. This may be due to replication fork DNA structures and associated repair complexes may undergo remodeling after prolonged stalling, leading to partial dissociation or reduced stability of these protein–protein interactions. Alternatively, it is possible that after assembling the MMEJ repair components at the stressed replication fork, RPRD1B is no longer needed, and these repair components can interact as a complex with the stressed fork on their own.

As our data shown above indicated that RPRD1B localization to stressed replication forks is RPA-dependent (Supplementary Fig. S4G), we next examined whether the interaction between RPRD1A and RPA1 is also DNA-dependent. The Co-IP data showed that the interaction between RPRD1A and RPA1 did not survive DNase1 treatment (Supplementary Fig. S5D) and was also lost following RPRD1B depletion (Supplementary Fig. S5E). These findings suggest that RPRD1B may facilitate the assembly or stabilization of RPRD1A–RPA1-associated complexes on RPA-coated DNA at stressed replication fork-associated repair machinery.

To assess the functional consequence of RPRD1B depletion on replication fork dynamics, we performed DNA fiber assays. While BRCA1-def cells have constitutive replication stress by virtue of their lack of HR repair, we analyzed the role of RPRD1B in these cells under added exogenous replication stress with hydroxyurea (HU) to assess their potential synthetic lethal vulnerabilities. Consistent with a role in fork repair, we found that loss of RPRD1B impair fork restart and progression in MDA-MB-436 BRCA1-def cells (Fig. 4D–G). Stalled replication forks were increased by three-fold in RPRD1B-depleted cells at 60 min compared with control cells. Measuring the track length of restarted replication forks compared with those same forks’ track length before stress found that RPRD1B-depleted, restarted forks were two-fold less in length than comparable controls. This indicates that RPRD1B-deficient replication forks were either slower to restart or were dysfunctional after restart and therefore progressed slower. We repeated this assessment in BRCA1-def HCC1937 cells and found qualitatively the same results. RPRD1B depletion resulted in a 1.4-fold increase in stalled replication forks at an earlier time point, 30 min (Supplementary Fig. S8A). Measuring the track length ratios of the pre-stress versus restarted forks found that RPRD1B depletion resulted in restarted forks that were two-fold less in length relative to their pre-stressed track lengths (Supplementary Fig. S8A). We also found that BRCA1-WT MCF7 cell replication forks did not require RPRD1B to recover from replication stress (Supplementary Fig. S8B). RPRD1B was not required for BRCA1-def HCC1937 cells to continue normal replication without stress (Supplementary Fig. S8C). Since the loss of RPRD1B resulted in the phosphorylation of multiple stressed fork repair components (Fig. 3A and B) without added replication stress, these activating phosphorylation events must be sufficient for maintaining replication fork progression when the BRCA1-def cells is not stressed. RPRD1B becomes essential when there is a stressor causing replication fork stalling. Finally, we did not see any evidence of replication fork degradation in HCC1937 cells after RPRD1B depletion in the presence of HU stress (Supplementary Fig. S8D).

### RPRD1B is required for efficient MMEJ and mitotic chromosomal segregation

**Figure 5.**
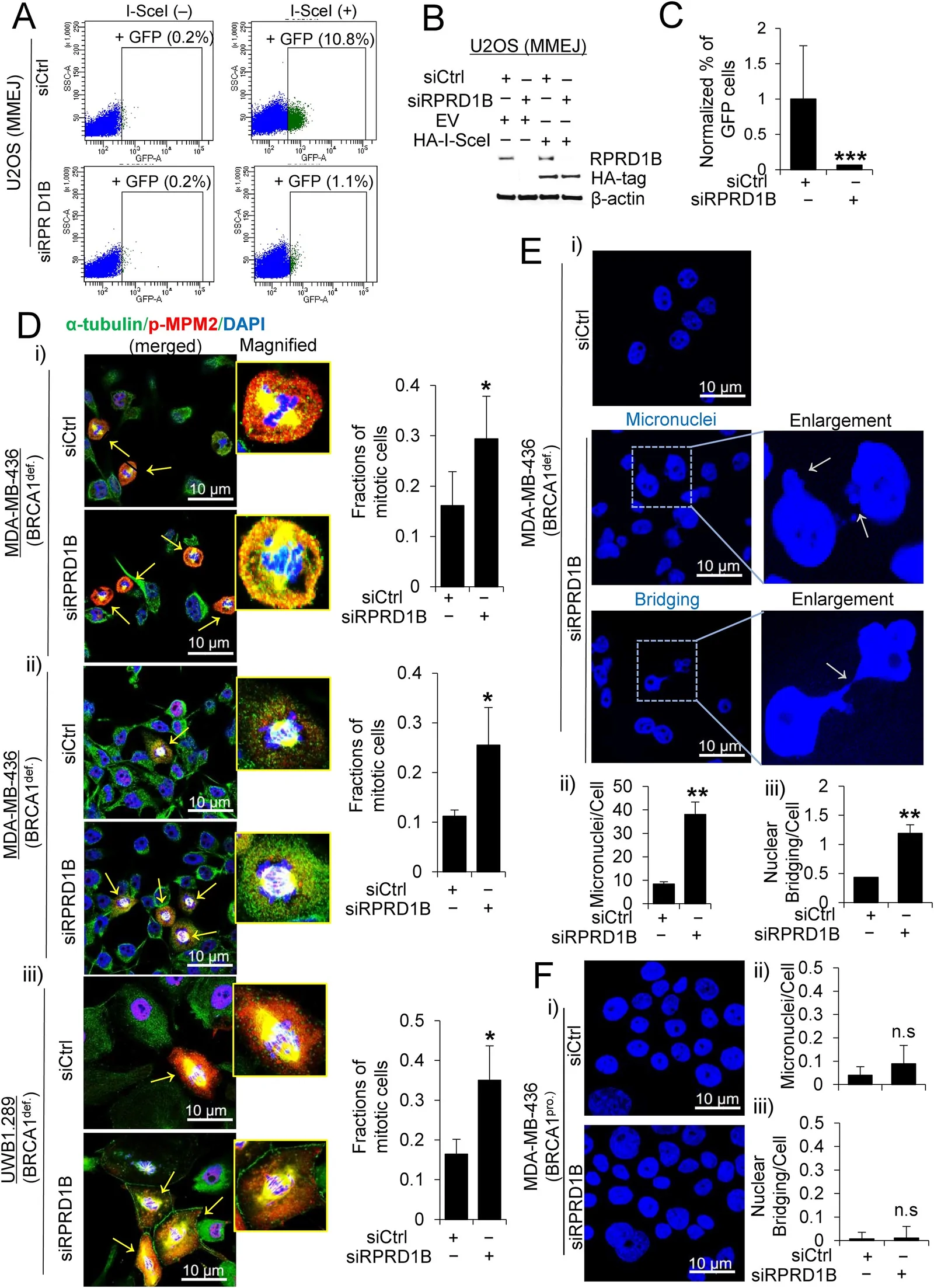
RPRD1B depletion reduces MMEJ activity and induces mitotic catastrophe in BRCA1-def cells. (**A**–**C**) RPRD1B depletion reduces MMEJ. U2OS EJ2 reporter cells were transfected with either siCtrl or siRPRD1B. After 24 h, cells were further transfected with either empty vector (EV) or HA-I-SceI plasmid. (**A**) Representative images of flow histograms of the EJ2 cells with and without I-SceI transduction. (**B**) The knockdown efficiency of RPRD1B and the expression of HA-I-SceI were confirmed by WB analysis. (**C**) Quantification of GFP-positive cells, indicating productive MMEJ repair, as measured by flow cytometry. Approximately 30 000 cells were analyzed per condition. Data are presented as mean ± SD from three independent experiments (*n* = 3). (**D**) (i) Representative confocal immunofluorescence images showing increased mitotic arrest in control (Ctrl) and RPRD1B-depleted BRCA1-def MDA-MB-436, HCC1937, and UWB1.289 cells stained with α-tubulin (green), p-MPM2 (red), and DAPI (blue). Arrows indicate arrested cells, most in anaphase. (ii) Quantification of mitotic cell fractions in siCtrl- or siRPRD1B-transfected cells. At least 100 cells were analyzed per condition. Data are shown as mean ± SD from five independent experiments (*n* = 5). *P ≤ .05. Scale bar, 10 µm. (**E, F**) Representative DAPI-stained confocal images showing micronuclei and nuclear bridging indicated by arrows, in siCtrl- or siRPRD1B-transfected BRCA1-def MDA-MB-436 cells (Ei-Eii) or BRCA1-pro MDA-MB-436 cells (Fi-Fiii). Quantification of percentage of cells with micronuclei and bridges (*n* = 5). At least 100 nuclei were analyzed per condition. **P ≤ .01; n.s, not significant. Scale bar, 10 µm.

BRCA1-def cells use MMEJ to repair and restart stressed replication forks, and inhibitors of MMEJ components, such as PARP1 and POLQ, can induce lethality in these cells [5–10, 34]. Since RPRD1B interacts with both PARP1 and POLQ *in vivo*, we asked whether RPRD1B is important for MMEJ efficiency, as it could explain its requirement for the survival of BRCA1-def but not -pro cells. Using the EJ2 reporter cell line, which uses MMEJ to repair a DSB to activate a green fluorescent protein (GFP) reporter [35], we measured MMEJ after depletion of RPRD1B (Fig. 5A–C). We found that RPRD1B depletion results in markedly reduced MMEJ DSB repair (10-fold decrease in Fig. 5C), providing the first evidence that RPRD1B is essential for MMEJ DSB repair.

To determine whether RPRD1B also regulates classical non-homologous end joining (cNHEJ), we next used the EJ5 reporter cell line, which measures cNHEJ-mediated DSB repair through restoration of GFP expression [35]. In contrast to the marked reduction of MMEJ activity, depletion of RPRD1B did not significantly alter cNHEJ repair efficiency (Supplementary Fig. S6A), suggesting that RPRD1B preferentially promotes MMEJ rather than canonical NHEJ repair.

Unrepaired stressed replication forks can lead to three main types of aberrant fork outcomes: (i) fork degradation, (ii) under-replicated DNA, or (iii) fusion of two forks at their free double-strand ends, which can occur from fork reversal or cleavage, both intermediate steps in repair of stressed replication forks [20, 36–40]. Unreplicated regions and chromosomal fusions can both lead to difficulty in segregating chromosomes during mitosis. Therefore, we assessed progression through mitosis in BRCA1-def cells after depletion of RPRD1B (Fig. 5D). We found that loss of RPRD1B generates a large fraction of cells arrested in mitosis at anaphase (1.8-fold increase in Fig. 5Di, 2.3-fold increase in Fig. 5Dii, and 2.1-fold increase in Fig. 5Diii), indicative of difficulty in chromosomes segregation, similar to the reconstitution of miR- 4485-3p in BRCA1-def cells. In contrast, there was no significant increase in mitotic arrest in the BRCA1-pro cells when RPRD1B was depleted (1.1-fold increase in Supplementary Fig. S6B, 1.2-fold increase in Supplementary Fig. S6C, and 1.2-fold increase in Supplementary Fig. S6D). Consistent with these findings, cell cycle analyses demonstrated that depletion of RPRD1B resulted in marked G2/M arrest in BRCA1-def cells, whereas BRCA1-pro cells and BRCA1-WT MCF7 cells did not exhibit significant alterations in cell cycle distribution under the same conditions (Supplementary Fig. S6E).

Unreplicated regions in chromosomes can lead to sister chromatids sharing one centromere, while fusion of broken replication forks can result in dicentric chromosomes, shared between daughter cells post-mitotically [7, 20, 28, 33]. The incompletely replicated sister chromatids can result in retained chromosomes seen as post-mitotic micronuclei, while dicentric chromosomes may be detected as a post-mitotic bridge between daughter cells [20, 28, 33]. Consistent with this, we found that the depletion of RPRD1B results in an increase in micronuclei (4.6-fold increase in Fig. 5Eii) and nuclear bridging (2.8-fold increase in Fig. 5Eiii) between daughter cells in the BRCA1-def cells but not the -pro daughter cells (∼1.2-fold increase in Fig. 5Eii and ∼1.1-fold increase in Fig. 5Fiii).

### RECQL5 recruits RPRD1B to stressed replication forks

**Figure 6.**
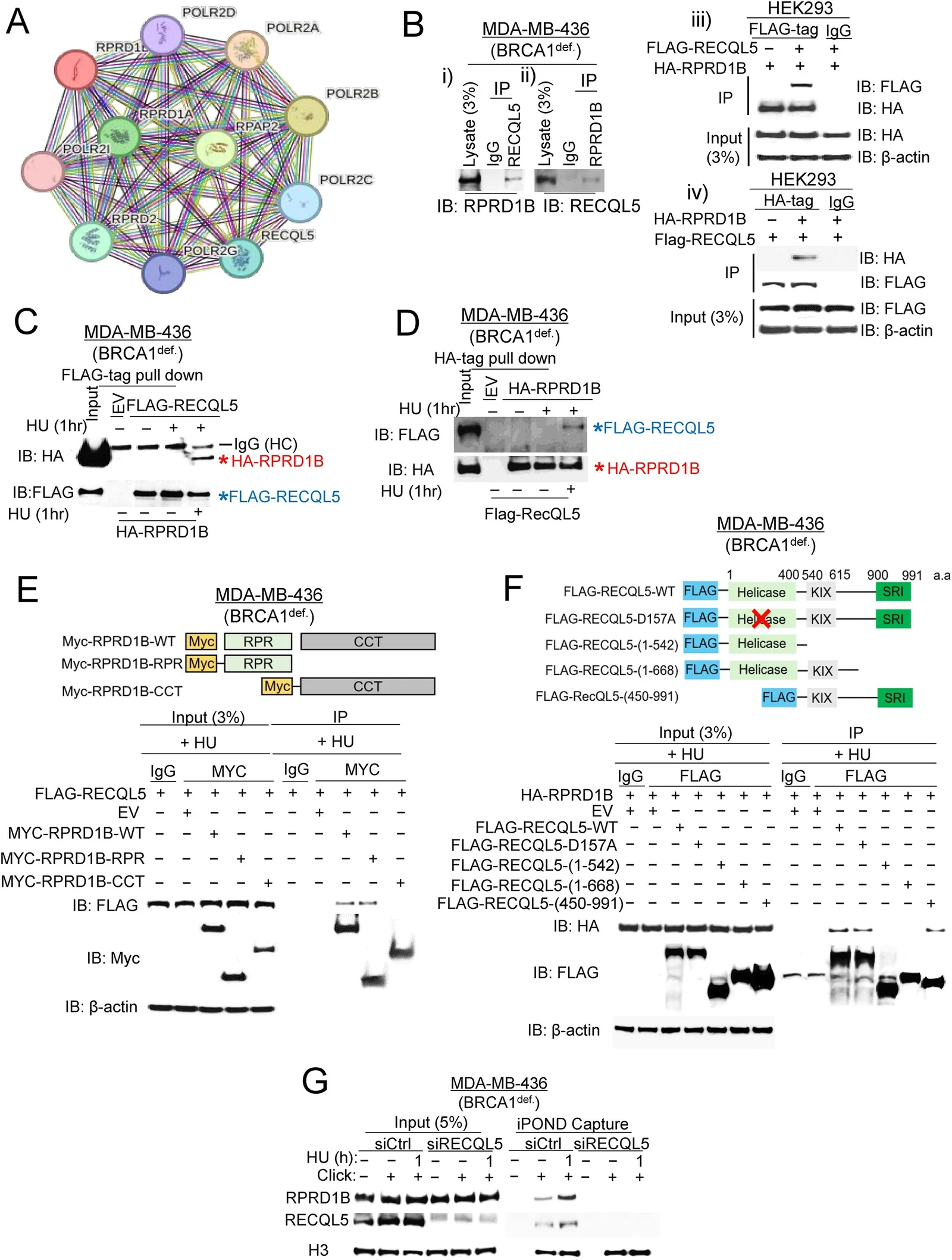
RECQL5 is essential for RPRD1B enrichment at stressed replication forks in BRCA1-def cells. (**A**) Protein–protein interaction network of RPRD1B generated using the STRING v9.1 database illustrating an interaction between RECQL5 and the RPRD family proteins including RPRD1B [26]. The network includes both predicted and experimentally validated interactions. Nodes represent Individual proteins, and lines indicate protein–protein associations. Line colors correspond to the type of supporting evidence: pink = experimental data, blue = curated database information, and green = co-expression. (**B**) RECQL5 and RPRD1B co-immunoprecipitate in BRCA1-def MDA-MB-436 cells and BRCA1 WT HEK293 cells. Endogenous Co-IP with antibodies against RECQL5 or normal rabbit IgG (i), and antibodies against RPRD1B or normal rabbit IgG (ii) were used to pull down proteins, followed by WB analyses using the indicated antibodies to detect associated proteins. Cell lysates prepared from HEK293 cells transiently expressing HA-RPRD1B with or without transfected FLAG-RECQL5 (iii), or FLAG-RECQL5 with or without transfected HA-RPRD1B (iv), were used for Co-IP analyses. In (iii), antibodies against HA or normal mouse IgG were used to pull down proteins, followed by WB analysis using the indicated antibodies to detect associated proteins. In (iv), antibodies against FLAG or normal mouse IgG were used to pull down proteins, followed by WB using the indicated antibodies to detect associated proteins. (C, D) *In vitro* direct interaction between FLAG-RECQL5 and HA-RPRD1B in BRCA1-def MDA-MB-436 cells. Cells were transfected with siRNAs targeting RPRD1B or RECQL5 to prevent isolation of the complex, followed by transfection with FLAG-RECQL5 or HA-RPRD1B, respectively. After treatment with or without 5 mM HU for 1 h, cell lysates were incubated with FLAG or HA beads at 4°C overnight (o/n). (**C**) Purified HA-RPRD1B was added to FLAG-RECQL5–bound beads, followed by washing and FLAG immunoprecipitation. (**D**) Purified FLAG-RECQL5 was incubated with HA-RPRD1B-bound beads, followed by washing and HA immunoprecipitation; bound FLAG-RECQL5 was detected by WB. (**E**) Domain-mapping analysis showing that the RPR domain of RPRD1B, but not the CCT domain, interacts with RECQL5. Myc-tagged RPRD1B and FLAG-tagged RECQL5 expression constructs were expressed in BRCA1-def MDA-MB-436 cells. Myc immunoprecipitation followed by WB was used to detect interaction. (**F**) RPRD1B preferentially interacts with the Set2-Rpb1-interacting (SRI) domain of RECQL5. HA-tagged RPRD1B and FLAG-tagged RECQL5 expression vectors were transfected into BRCA1-def MDA-MB-436 cells, FLAG immunoprecipitated, and then the immunoprecipitated was probed with anti-HA. WCLs were used, with ~3% loaded as input control. (**G**) RECQL5 is required for RPRD1B recruitment to stressed replication forks. BRCA1-def MDA-MB-436 cells were transfected with siCtrl or siRECQL5 and exposed to 5 mM HU for 1 h. iPOND analysis showed reduced RPRD1B enrichment at replication forks upon RECQL5 depletion. H3 served as a loading control.

Given the role of RPRD1B in the promotion of MMEJ at stressed replication forks, we next sought to define the mechanism by which RPRD1B is recruited to stressed forks, especially given the fact that RPRD1B does not possess a DNA-binding domain [22]. RECQL5 is a DNA helicase and RNAP2-binding protein thought to promote HR repair and also resolve replication stress by minimizing the level of R-loops [41–44]. The Biogrid database features RECQL5 as an interacting protein with RPRD1B (Fig. 6A), likely due to the presence of RECQL5 in an RPRD1B pulldown and mass spectroscopy analysis [26]. Based on these findings, we investigated whether RECQL5 might recruit RPRD1B to stressed replication forks in BRCA1-def cells. We found that endogenous RPRD1B co-immunoprecipitates with endogenous RECQL5 in BRCA1-def cells (Fig. 6B). We also demonstrated that transfected HA-tagged RPRD1B and Flag-tagged RECQL5 co-immunoprecipitated in BRCA1-WT HEK293 cells (Fig. 6B).

We purified HA-RPRD1B and Flag-RECQL5 from HU-stressed BRCA1-def cells. We found that each was able to pull down the other *in vitro* in Co-IP experiments, demonstrating that they interact directly (Fig. 6C and D). We then defined which domains of each protein mediate this interaction by generating expression vectors with deleted regions in RECQL5 and RPRD1B. We found that the RPR domain of RPRD1B, which mediates its interaction with RNA POL2, is also important for its interaction with RECQL5 (Fig. 6E) [45]. Moreover, we found that the SRI domain in RECQL5 is essential for its interaction with RPRD1B (Fig. 6F) [44, 46, 47]. We also assessed the direct interaction between RECQL5 and RPRD1B using purified proteins *in vitro* using an affinity pull-down assay with an anti-RECQL5 antibody and found that RECQL5 associates with RPRD1B (Supplementary Fig. S7A). We also used iPOND to test whether RECQL5 is important for RPRD1B recruitment to stressed replication forks in BRCA1-deficient cells after HU stress. Depletion of RECQL5 results in loss of RPRD1B recruitment to the stressed replication fork (Fig. 6G). These data imply that RECQL5 is essential for RPRD1B’s recruitment to the stressed replication fork.

### Ubiquitinated PCNA promotes RECQL5-RPRD1B localization to stressed replication forks

**Figure 7.**
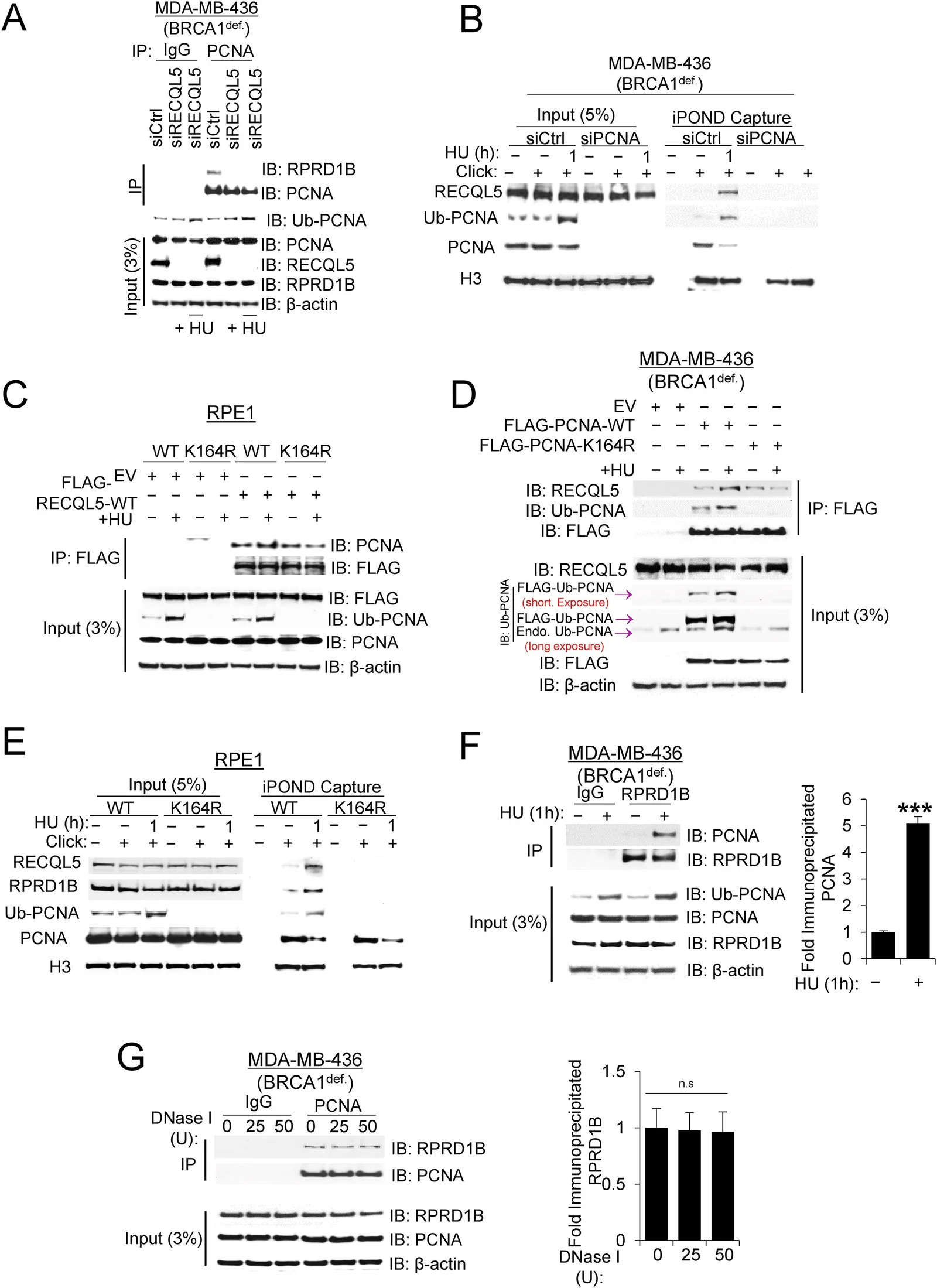
Ub PCNA is required to recruit the RECQL5-RPRD1B complex to stressed replication forks in BRCA1-def cells. (**A**) RECQL5 is required for the immunoprecipitation of RPRD1B with PCNA. BRCA1-def MDA-MB-436 cells were treated with siCtrl or siRPRD1B, followed by 5 mM HU for 1 h. Cell lysates were used for Co-IP with anti-PCNA or control IgG antibodies. WB analyses were performed to detect specific proteins with the indicated antibodies. (**B**) PCNA is required for RECQL5 recruitment to stressed replication forks. BRCA1-def MDA-MB-436 cells were transfected with siCtrl or siPCNA and exposed to HU (5 mM, 1 h). iPOND analysis showed reduced enrichment at replication forks upon PCNA depletion. Ub-PCNA and Histone H3 served as a replication stress response marker and a loading control, respectively. (**C, D**) K164-Ub PCNA enhances its interaction with RECQL5. (i) BRCA1 WT RPE-1 cells stably expressing PCNA*^WT^* or PCNA*^K164R^* were transfected with EV or FLAG-RECQL5 vector, followed by treatment with 5 mM HU for 1 h. WCLs were subjected to Co-IP using FLAG magnetic beads, followed by WB analysis with the indicated antibodies. (**C**) BRCA1-WT RPE-1 cells stably expressing PCNA*^WT^* and PCNA*^K164R^* were transfected with FLAG-PCNA-WT or FLAG-PCNA-K164R vectors, followed with or without HU treatment (5 mM, 1 h). Cell lysates were subjected to Co-I using anti-FLAG magnetic beads, followed by WB analysis with the respective antibodies. WCLs were used, with ~3% loaded as input control. (**D**) BRCA1-def MDA-MB-436 cells were transfected with EV, FLAG-PCNA-WT, or FLAG-PCNA-K164R vectors, followed by HU treatment (5 mM, 1 h). Cell lysates were subjected to Co-IP using anti-FLAG magnetic beads, followed by WB analysis with the respective antibodies. WCLs were used, with ~3% loaded as input control. (**E**) K164-Ub-PCNA promotes recruitment of RECQL5-RPRD1B complex to the stressed replication fork in BRCA1 WT RPE1 cells. RPE-1 cells stably expressing PCNA*^WT^* and PCNA*^K164R^* were exposed to 5 mM HU for 1 h. iPOND analysis was carried out to access recruitment of RECQL5 and RPRD1B to replication fork. Ub-PCNA and H3 served as a replication stress response marker and a loading control, respectively. (**F**) RPRD1B preferentially co-immunoprecipitates with Ub-PCNA in BRCA1-def MDA-MB-436 cells after stress. BRCA1-def MDA-MB-436 cells were exposed to 5 mM HU for 1 h. Cell lysates were subjected to Co-IP using antibodies against normal rabbit IgG or RPRD1B to pull down proteins. WB analysis (left) was performed using the indicated antibodies. Densitometric quantification of immunoprecipitated PCNA compared to RPRD1B with or without HU treatment is shown (right). (*n* = 3, means with ± SD). ***P ≤ .001. WCLs were used, with ~3% loaded as input control. (**G**) The immunoprecipitation of RPRD1B with Ub-PCNA is DNA-independent. BRCA1-def MDA-MB-436 cells were exposed to 5 mM HU for 1 h. Cell lysates were treated with DNase I (0, 25, or 50 U) for 30 min at 37°C, followed by Co-IP using anti-PCNA or control rabbit IgG antibodies. WB analyses (upper) using anti-RPRD1b was performed. Quantification of RPRD1B immunoprecipitated with PCNA (lower graph). (*n* = 3). n.s, not significant. WCLs were used, with ~3% loaded as input control.

Interestingly, while the previous proteomic analysis of RPRD1B interactions found that PCNA is an interacting partner [26], we could not observe a direct RPRD1B interaction *in vitro* with either PCNA or ubiquitinated (Ub) PCNA, examined with a TALON bead pull-down assay when these purified proteins are alone together (Supplementary Fig. S7A). RPRD1B does not have a PIP box or non-canonical PCNA-interacting sequences, while RECQL5 possesses both a PIP box (in the SRI domain) as well as a PIP-L domain and has been reported to interact with PCNA [41–44]. We therefore asked whether the previously published [26] *in vivo* Co-IP of RPRD1B and PCNA was via RECQL5, and that PCNA promoted recruitment of RECQL5-RPRD1B to stressed replication forks. We found that RECQL5 is indeed important for Co-IP of PCNA with RPRD1B (Fig. 7A), and we also confirmed a direct interaction between RECQL5 and RPRD1B by *in vitro* pull-down assay (Supplementary Fig. S7B), implying that RECQL5 may serve as a bridging factor mediating bridges the PCNA–RPRD1B interaction observed in the prior proteomic analyses reporting an interaction between RPRD1B and PCNA.[26] Although RECQL5 can bind DNA on its own [41–44], we investigated whether PCNA would promote the recruitment of RECQL5 to the stressed replication forks. We found by iPOND analysis that depletion of PCNA leads to diminished recruitment of RECQL5 to stressed replication forks in these cells (Fig. 7B). Since PCNA is Ub at K164 after replication stress and this promotes recruitment of translesion synthesis proteins to stalled replication forks, we investigated whether RECQL5 could still co-immunoprecipitate with PCNA if K164 in PCNA is mutated to arginine [41–44, 48, 49], which cannot undergo either sumoylation or ubiquitination upon occurrence of replication stress. We found that RECQL5 co immunoprecipitates less well with PCNA K164R than WT Ub-PCNA (Fig. 7C and D), and RECQL5 recruitment to stressed forks becomes attenuated in cells that express this PCNA mutant as determined by iPOND (Fig. 7E).

**Figure 8.**
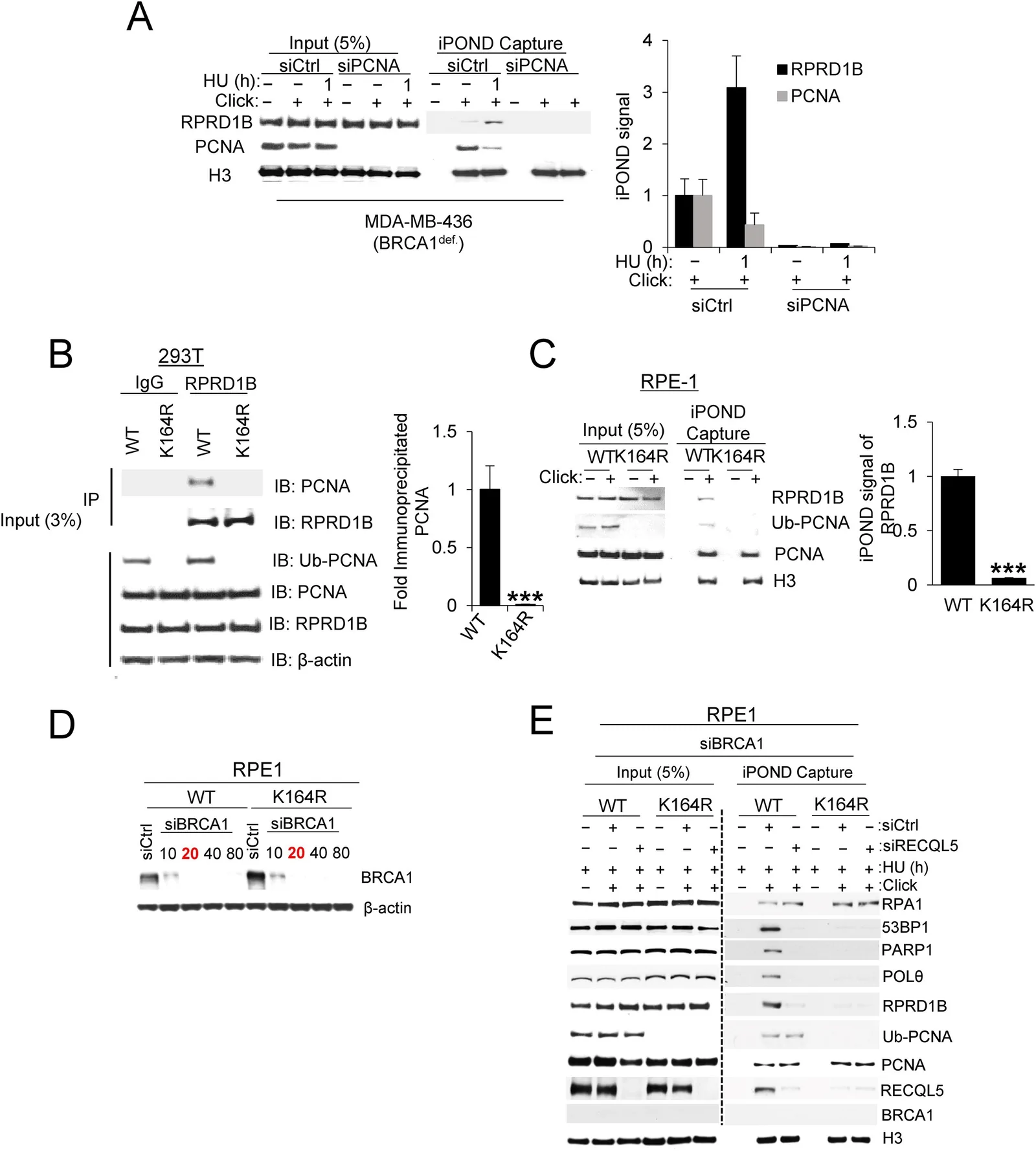
RECQL5-dependent recruitment of RPRD1B and fork repair factors at stressed replication forks downstream of BRCA1 and PCNA ubiquitination. (**A**) RPRD1B recruitment to the stressed replication fork in BRCA1-def MDA-MB-436 cells is dependent on PCNA. Cells were treated with siCtrl or siPCNA and then exposed to 5 mM HU for 1 h. iPOND analysis was performed to access recruitment of RPRD1B to replication forks. Ub-PCNA and H3 served as a replication stress response marker and a loading control, respectively (left). Densitometric quantification of iPOND signals, *n* = 3 (right). (**B**) RPRD1B preferentially immunoprecipitates with Ub-PCNA. Cell lysates from 293T cells stably expressing PCNA*^WT^* and PCNA*^K164R^* were prepared for Co-IP using anti-RPRD1B or control rabbit IgG antibodies. WB analysis (*left*) was performed with the indicated antibodies. Densitometric quantification of PCNA immunoprecipitated with RPRD1B is shown (right). (*n* = 3, means with ± SD). ***P ≤ .001. WCLs were used, with ~3% loaded as input control. (**C**) Ub-PCNA recruits RPRD1B to stressed replication forks. BRCA1 WT RPE-1 cells stably expressing PCNA*^WT^* and PCNA*^K164R^* were analyzed by iPOND to access RPRD1B recruitment to replication forks. Ub-PCNA and H3 served as a replication stress response marker and a loading control, respectively (left). Densitometric quantification of iPOND signals is shown (right). (*n* = 3). ***P ≤ .001. (**D**) WB analysis showing BRCA1 protein depletion following transfection with siCtrl or increasing concentrations of siBRCA1 (0, 10, 20, 40, and 80 nM) in BRCA1 WT RPE-1 cells stably expressing PCNA*^WT^* and PCNA*^K164R^* RPE-1 cells to determine the optimal knockdown condition for downstream iPOND analysis. (**E**) iPOND analysis showing RECQL5-dependent recruitment of RPRD1B and replication fork repair factors (PARP1, POLQ, and 53BP1) at stressed replication forks in BRCA1-depleted RPE-1 cells stably expressing PCNA*^WT^* and PCNA*^K164R^* RPE-1 cells. Ub-PCNA and H3 served as a replication stress response marker and a loading control, respectively.

We also found that RPRD1B co-immunoprecipitated with PCNA upon treatment with HU (Fig. 7F). This interaction is not affected by DNase1 addition, indicating that complex formation can occur without replication fork structures (Fig. 7G). If RECQL5 were recruited to the stressed replication fork by PCNA, then RPRD1B should also be recruited to the stressed fork by PCNA via RECQL5. We therefore depleted PCNA from BRCA1-def cancer cells and found that the recruitment of RPRD1B to stressed replication forks becomes impaired (Fig. 8A). These data imply that Ub-PCNA is crucial for the recruitment of RECQL5 and RPRD1B to stressed replication forks, at least in BRCA1-def cells [48]. Finally, we asked whether the Co-IP of RPRD1B with PCNA could survive the mutation of K164 to R in PCNA. Consistent with our findings for RECQL5, we demonstrated that RPRD1B neither co-immunoprecipitates with PCNA K164R (Fig. 8B; Supplementary Fig. S7C and D) nor is recruited to stressed replication forks (Fig. 8C) [48].

To further investigate the functional importance of Ub-PCNA-mediated RECQL5 recruitment, we performed iPOND analyses in WT-PCNA and PCNA K164R RPE1 cells following BRCA1 depletion, with or without RECQL5 depletion (Fig. 8D,E). Interestingly, PCNA K164R markedly impaired recruitment of RECQL5, RPRD1B, POLQ, 53BP1, and PARP1 to stressed replication forks, while depletion of RECQL5 similarly reduced recruitment of RPRD1B, POLQ, 53BP1, and PARP1 (Fig. 8E). In contrast, RECQL5 depletion did not appreciably affect the abundance of RPA1 or endogenous PCNA at stressed replication forks (Fig. 8E). These findings support a model in which Ub PCNA promotes RECQL5-dependent recruitment of RPRD1B-associated repair complexes to stressed replication fork as shown in the graphical abstract.

## Discussion

This study uncovers a new synthetic lethality relationship in BRCA1-def cells through the assembly of the MMEJ machinery via the scaffolding function of RPRD1B. Discovery of the synthetic lethality prevented by RPRD1B was the result of a persistent exploration of miR repressed in BRCA1-def cancer cells. Loss of BRCA1 results in activated IRE1 repressing a cohort of miRNAs that would otherwise affect the fitness of the BRCA1-def cells, permitting their survival for potential neoplastic transformation [19–22]. Without the repression of these miRNAs, BRCA1 loss would be lethal. As it is, this cohort of miRNAs has served as a productive template from which to define novel synthetic lethal relationships in BRCA1-def cancer cells, by identifying the miRNAs’ targets.

We have demonstrated that miR-4485-3p decreases translation of RPRD1B, and that RPRD1B promotes survival of BRCA1-mutant cells by recruiting multiple MMEJ components, 53BP1, PARP1, and POLQ to stressed replication forks [8–11, 13, 26]. POLQ plays an essential role in MMEJ, both promoting the annealing at microhomologies and then synthesis from the free ends [8–11]. Thus is it not surprising that loss of RPRD1B blocks MMEJ, since it decreases POLQ recruitment to the stressed BRCA1-def replication fork, resulting in increased unrepaired replication forks after stress in BRCA1-def cells. Consistent with this dependency, components of the MMEJ pathway, including FEN1, POLQ and PARP1 have been implicated in replication-associated repair processes required for genome maintenance in BRCA-def cells. Loss or inhibition of FEN1 has been reported to impair survival of HR-def cells, supporting the concept that MMEJ-associated end processing enzymes represent functional vulnerabilities in this context [16–18].

Interestingly, loss of RPRD1B resulted in significantly decreased replication fork track length after recovery from stress in both BRCA1-def cell lines examined. This implied that either the restarted replication fork was defective in progression or that restart itself was slower. We and others have found that even a short increase in the time for replication fork restart after stress can result in cell death [27, 28]. This increase in time for replication completion can result in cells entering mitosis before all DNA is replicated, and can result in the chromosomal segregation failure we saw in these cells with loss of RPRD1B. However, we did not see any evidence of replication fork degradation in HCC1937 cells after RPRD1B depletion in the presence of HU stress. This implies that RPRD1B loss does not inhibit recruitment of the main stressed replication fork protection components, such as ABRO1 or BRCA2 and these components are able to protect the stalled replication forks after RPRD1B depletion [50, 51]. This is not surprising since these fork protection proteins are not part of the MMEJ repair apparatus.

Although RPRD1B depletion reduced RAD51 recruitment to stressed replication forks, the functional contribution of RAD51 to the RPRD1B-dependent repair pathway remains to be fully defined. RAD51-mediated SS DNA and/or fork protection can occur through mechanisms distinct from BRCA1/2 and canonical HR.[50–53] Since the primary objective of the present study was to define the role of RPRD1B in promoting MMEJ-associated repair through recruitment of 53BP1, PARP1, and POLQ, further work will be needed to characterize the connection between RAD51 fork protection and RPRD1B scaffolding in BRCA1-deficient stressed replication forks.

In addition, although RPRD1B depletion did not significantly alter the proportion of stalled replication forks without HU stress, RPRD1B-def cells did exhibit increased basal levels of γH2AX, phosphorylated ATR, and phosphorylated RPA. We propose that RPRD1B primarily functions as a scaffolding factor that promotes the recruitment and assembly of repair components, including 53BP1, PARP1, and POLQ at stressed replication forks rather than directly preventing fork stalling. Therefore, depletion of RPRD1B may impair the resolution of spontaneous replication-associated lesions that arise during normal DNA replication in BRCA1-deficient cells without stress. This results in activation of ATR-mediated replication stress responses and accumulation of DNA damage markers without causing measurable alterations in bulk replication fork dynamics under the experimental conditions examined. Perhaps the bulk of replication forks proceeds without repairing the endogenous spontaneous DNA lesions when RPRD1B is lost, and this results in the genomic damage evidenced by the increased γ-H2Ax and the mitotic catastrophe. This interpretation is consistent with our model that RPRD1B-mediated assembly of the MMEJ repair machinery is required for efficient resolution of fork lesions after stress and maintenance of genome stability in BRCA1-def cells.

Importantly, this pathway is consistent with emerging therapeutic strategies targeting the MMEJ axis, as inhibition of POLQ has been shown to selectively sensitize BRCA1-def tumors by impairing alternative end-joining and exacerbating replication stress [54, 55]. Consistent with this therapeutic vulnerability, clinical and preclinical studies of POLQ inhibitors highlight the importance of MMEJ dependency in HR-deficient tumors and support the rationale for targeting this repair axis in cancer therapy [54, 56, 57]. Our findings extend this concept by identifying RPRD1B as an upstream scaffolding factor required for efficient assembly of POLQ-associated repair complexes at stressed replication forks. Thus, targeting the Ub-PCNA–RECQL5–RPRD1B signaling axis may provide an alternative strategy to disrupt POLQ-dependent repair and induce synthetic lethality in BRCA1-deficient cancers, upstream of POLQ.

We have also shown that this failure of MMEJ and increase in unrepaired stressed replication forks lead to catastrophic mitotic events, including micronuclei and chromosomal bridging. This confirms the link between unrepaired stressed replication forks, aberrant chromosomal segregation, and subsequent mitotic failure [20, 28, 33]. This mitotic catastrophe likely underpins the mechanism of cell death in the BRCA1-def cancer cells when RPRD1B is depleted.

Our data indicate that RPRD1B may initiate MMEJ repair by recruiting other MMEJ components to stressed replication forks. Given that RPRD1B does not possess a classic DNA-binding domain [22], it is likely recruited to stressed replication forks via interaction with another DNA binding factor. In this regard, our proteomic analysis of the RPRD1B immunoprecipitate identified RECQL5, known to play a role in stressed replication fork repair and restart as a potential mediator of RPRD1B replication fork recruitment [41–44]. Our molecular analysis confirmed that RECQL5 and RPRD1B interact directly, and this interaction is enhanced by replication stress.

Interestingly, our temporal iPOND and Co-IP analyses suggest that RPRD1B recruitment and its associated repair complexes during the early phase of HU-induced replication stress (1 h) but decreases following prolonged HU exposure (2 h). These findings suggest that RPRD1B may function transiently during the initial stages of stalled replication fork recognition and repair complex assembly, after which the assembled repair complex can interact with the fork structures on its own. Such temporal regulation is consistent with the dynamic organization of replication stress repair components at the fork [58, 59].

Notably, both RECQL5 and RPRD1B bind to the C-terminus of RNAP2 and regulate its activity [43, 45, 46]. RECQL5 decreases RNAP2 transcription to resolve R-loops for the avoidance of DNA replication conflicts, and RPRD1B could enhance that RECQL5 activity as well [43, 47, 60]. Such synergy would be especially important in BRCA1-def cancers, as these cells exhibit increased transcription-replication collisions because BRCA1 normally helps resolve R-loops [61, 62]. We also previously found that R-loop resolution could be a synthetic lethal weakness of BRCA1-def cells when TATDN2, a DNA:RNA-specific RNase was depleted [61]. Future studies to elucidate the role of the RECQL5-RPRD1B complex in preventing transcription-replication conflicts may shed further light on the mechanism of the slower restart and progression of stressed replication forks after RPRD1B depletion.

Our finding that RECQL5 directly interacts with RPRD1B, and that both proteins associate with Ub PCNA, suggests that this complex may serve as an initiating platform for MMEJ recruitment at stressed replication forks, which is required for survival when exogenous replication stress is added. Thus, this study provides a more complete understanding of the molecular cascade underlying MMEJ-mediated repair of stressed replication forks. Specifically, Ub-PCNA recruits RECQL5, which in turn recruits RPRD1, leading to assembly of downstream MMEJ factors including 53BP1, PARP1, and POLQ. 53BP1 limits extensive DNA end resection, thereby favoring end-joining repair. In BRCA1-mutant cells, progression toward HR pathway may be deleterious because these cells cannot complete HR repair efficiency.

The role of PCNA in recruiting RECQL5 and then RPRD1B as an initial step in MMEJ repair of stressed replication forks in BRCA1-mutant cells indicates a previously unidentified role for Ub PCNA in the preservation of stressed replication forks [62, 63]. Consistent with this, PCNA is crucial for alternative lengthening of telomeres, a process similar to MMEJ [62].

Collectively, this study describes a previously unrecognized Ub-PCNA–RECQL5–RPRD1B–POLQ/PARP1 signaling axis that is essential for replication fork repair and survival of BRCA1-def cancer cells (modeled in Fig. 8C). It is possible that this pathway may also be responsible for replication stressing chemotherapy, such as with the TOP1 inhibitors or cisplatin, agents commonly used for treating BRCA1-def cancers [64, 65]. Thus, therapeutic targeting of this pathway may therefore provide a promising strategy to induce synthetic lethality in BRCA1-def tumors, particularly in patients who have developed resistance to PARP inhibitors.

## Supplementary Material

gkag831_Supplemental_Files

## Data Availability

The original data of this report are uploaded to the Mendeley site: https://data.mendeley.com/preview/h92sg7rnkj and Figshare at https://doi.org/10.6084/m9.figshare.33109475.
