# Supplementary material for: PCNA–RECQL5–RPRD1B recruit repair components to stressed replication forks to promote survival of BRCA1-deficient cancer cells
Source: Nucleic Acids Res. 2026 Aug 27;54(16):gkag831. doi: 10.1093/nar/gkag831 (PMC13519111; doi:10.1093/nar/gkag831)
Supplement: Supplementary file 1 [file gkag831_supplemental_files.zip › Supplementary Data Extended Methods and Materials.docx]

# **SUPPLEMENTAL METHODS AND MATERIALS**

*The original data of this report is uploaded to the Mendeley site: https://data.mendeley.com/preview/h92sg7rnkj.*

**Cell Culture**

Human cell lines used in this study included BRCA1-deficient (def) MDA-MB-436 (breast cancer), BRCA1-prolete(rep) MDA-MB-436, BRCA1-def and BRCA1-pro HCC1937 (breast cancer), BRCA1-def and BRCA1-pro UWB1.289 (ovarian cancer), BRCA1-wild type (WT) MCF7 (breast cancer), HEK293 and HEK293T (embryonic kidney), RPE-1 (TERT-immortalized retinal epithelium) and EJ2-GFP U2OS and EJ5-GFP U2OS (osteosarcoma). BRCA1-reconstituted (BRCA1-proficient) MDA-MB-436 [1] and HCC1937 [2] cells were generated previously by stable expression of full-length wild-type BRCA1 in the corresponding BRCA1-deficient parental cell lines. Briefly, cells were transfected with a plasmid encoding wild-type BRCA1 and subsequently subjected to G418 selection to establish stable clones. BRCA1 reconstitution was confirmed by Western blot analysis demonstrating restored BRCA1 protein expression in the reconstituted cells. The HEK293 and U2OS cell lines were cultured in Dulbecco’s Modified Eagle’s Medium (DMEM) supplemented with 10% fetal bovine serum (FBS), 1% penicillin/streptomycin and maintained at 37^o^C in a humidified incubator with 5% CO_2_. The EJ2-GFP and EJ5-GFP U2OS cells were a gift from Dr. Jeremy Stark (City of Hope, Duarte). BRCA1-def and BRCA1-pro MDA-MB-436 cells were cultured in DMEM medium supplemented with 10% FBS, 1% penicillin/streptomycin at 37^o^C with 5% CO_2_, and HCC1937 cells were cultured in RPMI 1640 medium supplemented with 10% FBS, 1% penicillin/streptomycin under the same incubation conditions. BRCA1-def and BRCA1-pro UWB1.289 (ovarian cancer) were cultured in 1:1 mixture of RPMI 1640 and MEGM medium, supplemented with 6% FBS and 1% penicillin/streptomycin, also at 37^o^C with 5% CO_2_. HEK293T (*PCNA^WT^* and *PCNA^K164R^*), and h-TERT RPE-1 [*PCNA^WT^*, *PCNA^K164R(A1)^*, *PCNA^K164R(B1)^* and *PCNA^K164R(2B10)^*] cells were cultured in DMEM supplemented with 10% FBS and 1% penicillin/streptomycin and incubated at 37^o^C with 5% CO_2_.

**MiRNA, siRNA, plasmids, and transfection**

MirVana miR-4485-3p microRNA mimic (Sequence: UAACGGCCGCGGUACCCUAA, Cat.# 4464066) was purchased from Thermo Fisher Scientific (Waltham, MA, USA). ON-TARGETplus SMARTpool small interfering RNAs (siRNAs) targeting RPRD1B (Cat.# L-013787-01-0020), RPRD1A (Cat.# L-007734-00-0005), RPA1 (Cat.# L-015749-01-0020) and RECQL5 (Cat.# L-0193380-00-0010) were purchased from Horizon. Additional siRNAs targeting RPRD1B (Cat.# 4392420) and PCNA (Cat. # 4390825) were obtained frm Thermo Fisher Scientific.

BRCA1-def. or -rep. MDA-MB-436, HCC1937,and UWB1.289, or BRCA1^wt^ MCF7, PCNA^wt^ or PCNA^K164R^ RPE1 cells were transiently transfected using Lipofectamine RNAiMAX reagent (ThermoFisher Scientific), according to the manufacturer’s instructions.

For transfection, cells were seeded 24 h prior to transfection at densities of 2 ×10^5^ cells/well in 6-well plates for colony formation assay (CFA), western blot (WB), or immunofluorescence (IF); 5 ×10^6^ cells in 10-cm dish for co-immunoprecipitation (Co-IP); or 2 ×10^7^ cells in 15-cm dishes for isolation of proteins on nascent DNA (iPOND) assays.

Transfection complexes were prepared by diluting 6 µL of RNAiMAX in 250 µL Opti-MEM medium. In parallel, miRNA mimic or siRNA was diluted in 250 µL Opti-MEM to final concentrations of 50 nM for miR-4485-3p mimic, 30 nM for RPRD1B siRNA, 40 nM for RPA1 siRNA, 40 nM for RECQL5 siRNA, and 20 nM for BRCA1 siRNA. The diluted RNA and Lipofectamine mixtures were combined and incubated at room temperature (RT) for 20 min before addition to cells.

Six hours after transfection, 0.5 mL of fresh medium containing 30% FBS was added to each well. The transfection medium was replaced with complete growth medium after 24 h. Cells were harvested 48 h after siRNA transfection or 72 h after miRNA transfection for CFA, WB, IF, Co-IP, or other indicated assays. All experiments were independently repeated at least three times.

**MicroRNA quantitation assay**

MiR-4485-3p expression was quantified using quantitative reverse transcriptase PCR as previously described. Global miR expression profiling following IRE1 depletion were performed using microarray technology performed by LC Sciences (Houston, TX).

Briefly BRCA1-def MDA-MB-436 cells were transiently transfected with either IRE1 siRNA or control siRNA (siCtrl) as described above. Total RNA was isolated forty-eight hours post-transfection from three independent biological replicates for each condition. The RNA samples were subsequently subjected to microRNA microarray by LC Sciences to determine differential miRNA expression profiles following IRE1 depletion. siRNAs used in this study and transfection procedures are described in the “miRNA, siRNA, plasmids, and transfection” section above.

**Luciferase assay for assessing RPRD1B regulation by miR-4485-3p**

One day prior to transfection, cells were seeded in 6-well plates at a density of 2 × 10^5^ cells per well. For microRNA transfection, cells were transfected with 50 nM control miRNA or miR-4485-3p mimic, using Lipofectamine RNAiMAX reagent (Thermo Fisher Scientific) according to the manufacturer’s instructions. miRNAs were transfected prior to reporter plasmid transfection to allow sufficient time for incorporation into the RNA-induced silencing complex (RISC) and establishment of miRNA-mediated repression before assessment of luciferase reporter activity.

Twenty-four hours after miRNA transfection, cells were transfected with 1.5 μg of RPRD1B 5’ UTRLuc-SV40-RenLuc-2A-Puro plasmid (Cat.# Custom-1, Applied Biological Materials, Inc.) using FuGENE6 transfection reagent (Promega), according to the manufacturer’s instructions.

Cells were lysed forty-eight hours after plasmid transfection (corresponding to seventy-two hours after miRNA transfection), and luciferase activity was measured using Luc-Pair^TM^ Dual Luciferase Assay Kit (Cat.# LP004-P010, GeneCopoeia), according to the manufacturer’s instruction. Firefly luciferase activity was normalized to Renilla luciferase activity. All assays were performed using three independent biological replicates, and each experiment was independently repeated at least three times.

**Colony formation assay**

Clonogenic assays were performed as previously described. Following microRNA or siRNA transfection, approximately 800 cells per well were seeded into 6-well plates and cultured for 2–3 weeks to allow colony formation. Colonies were then fixed with 4% paraformaldehyde for 20 min, stained with 0.1% crystal violet in methanol for 10 min, followed by rinsing with distilled water. Plates were air-dried and imaged. Colony numbers were quantified using ImageJ software (NIH).

Statistical analyses were performed using unpaired Student’s t tests unless otherwise specified. All experiments were conducted in three independent replicates and independently repeated at least three times.

**Ub-PCNA generation**

PCNA was expressed in *Escherichia coli* (*E. coli*) Rosetta cells (EMD Millipore) transformed with pET39-PCNA plasmid with 0.5 mM IPTG at 16°C for 16 h. Cell pellets (30 g) were resuspended in 200 ml buffer T (25 mM Tris-HCl, pH 7.5, 10% glycerol, 0.5 mM EDTA, 1 mM DTT, 0.01% Igepal CA-630) supplemented with 100 mM KCl and protease inhibitors (PIs) (1 mM PMSF and 2 μg/ml each of aprotinin, chymostatin, leupeptin, and pepstatin A). Cells were lysed by sonication, and the lysates were clarified by ultracentrifugation at 100,000 × *g* for 90 min at 4^o^C. PCNA was purified sequentially using a 25 ml-Fast-flow Q Sepharose column (GE Healthcare) with a 300 ml-gradient of 100 to 700 mM KCl in buffer T, followed by a 10 ml Macro hydroxyapatite column (Bio-Rad) with a 100 ml-gradient of 0 to 300 mM phosphate in buffer T, and finally a 1 ml Mono Q column (GE Healthcare) with a 50 ml gradient of 50–400 mM KCl in buffer T. PCNA-containing fractions from the Mono Q column were concentrated using Amicon Ultra-15 centrifugal filters (30 kDa MWCO; EMD Millipore) and stored at −80 °C.

His_6_-Uba1 was expressed in E. coli Rosetta cells transformed with pET-his_6_-Uba1 by induction with 0.1 mM IPTG at 16°C for 16 h. Cell pellets (9.5 g) were resuspended in 200 ml buffer K (20 mM KH2PO4; pH 7.5; 10% glycerol; 0.5 mM EDTA; 0.01% Igepal CA–630) supplemented with 300 mM KCl and PIs. Cell lysates were prepared as described above. The clarified lysate was incubated with 2 ml Talon Superflow (Sigma) and 20 mM imidazole for 2 h at 4^o^C. The resin was transferred to a column and washed with buffer 50 ml of buffer K containing 1000 mM KCl, and bound proteins were eluted with buffer K supplemented with 150 mM KCl and 200 mM imidazole. His_6_-Uba was further purified on a 1 ml HiTrap Q column (Cytiva) using a 14 ml-gradient of 100–500 mM KCl in buffer K. Peak fractions were concentrated using Amicon Ultra-15 centrifugal filter, aliquoted, and stored at −80 °C.

His_6_-UbcH5c-S22R was expressed in *E. coli* Rosetta cells transformed with pET28-UbcH5c-S22R plasmid (Addgene Cat.# 12644) by induction with 0.1 mM IPTG at 16°C for 16 h. Cell pellets (14 g) were resuspended in 200 ml buffer M (30 mM MES, pH 6.0, 1mM EDTA, 1 mM DTT, and PIs). Cells were lysed by sonication, and lysates were clarified by centrifugation at 18,000 × g for 30 min at 4^o^C. UbcH5c-S22R was purified using a 10 ml-Fast-flow SP Sepharose column (GE healthcare) with a 100 ml linear gradient of 0–500 mM NaCl in buffer M, followed by size-exclusion chromatography on a Superdex 200 10/30 (Cytiva) equilibrated in buffer T with 300 mM KCl. Peak fractions were concentrated using Amicon Ultra-15 centrifugal filters and stored at −80 °C.

Ubiquitin-conjugated PCNA (Ub-PCNA) was prepared using an *in vitro* mono-ubiquitination reaction as described previously with minor modifications. In a total reaction volume of 1,250 μL, purified PCNA (4 μM), UbcH5c-S22R (2 μM), and Uba1 (200 nM) and ubiquitin (20 μM, R&D systems Cat#: U-100H) were incubated in 35 mM Tris-HCl (pH 7.5), 1 mM DTT, 3 mM ATP, and 3 mM MgCl2 at 37°C for 90 min. Ub-PCNA was purified with a 1 ml HiTrap Q column using a 3 ml-stepwise gradient of 100, 200, 500 mM KCl in buffer T. The fraction eluted with 500 mM KCl was concentrated, aliquoted, and stored at −80 °C.

**His_6_-RPRD1B and RECQL5 protein purification**

For the Talon bead pull-down *in vitro*, the plasmid encoding His_6_-tagged RPRD1B was transformed into *E. coli* Rosetta cells (EMD Millipore). For large-scale expression, six 1 L cultures of LB medium were each inoculated with 50 mL of an o/n starter culture and incubated at 37^o^C, 180 rpm until the optical density at 600 nm (OD_600_) reached 0.9–1.0. Cultures were then incubated on ice for 1 h, followed by the addition of ethanol to a final concentration of 0.1%. Protein expression was induced by the addition of 0.3 mM IPTG, and cultures were grown at 18^o^C with shaking at 180 rpm for 18–20 h. Cells were harvested by centrifugation at 5,000 rpm for 10 min at 4^o^C and proceeded immediately for protein purification.

Cell pellets obtained from 6 L of LB culture were resuspended in 50 mL of lysis buffer per liter of culture (50 mM HEPES, pH 7.5, 300mM NaCl, 10% Glycerol). Prior to lysis, a protease inhibitor cocktail and Turbonuclease were added according to the manufacturer’s instructions. Cells were lysed by sonication at 30% amplitude for 4–5 min, until viscosity was reduced. Lysates were clarified by centrifugation at 20,000 rpm for 30 min at 4^o^C. The resulting supernatant for the HIS-RPRD1B was applied to a Ni-NTA gravity column (Quiagen) pre-equilibrated with lysis buffer. The Ni-NTA column was washed sequentially with lysis buffer and wash buffer containing 50 mM HEPES, pH 7.5, 300 mM NaCl, 10 % Glycerol, and 20 mM Imidazole. Bound proteins were eluted with 30 mL of elution buffer consisting of 50 mM HEPES, pH 7.5, 300 mM NaCl, 10 % Glycerol, and 250 mM Imidazole. The pooled Ni-NTA eluates were diluted to adjust the final NaCl concentration to 60 mM and subsequently loaded onto a 5 mL HiTrap Heparin column (Cytiva) equilibrated with Heparin Buffer A (50 mM HEPES, pH 7.5, 50 mM NaCl, 5% Glycerol) at a flow rate 1.5 mL/min. The column was washed with 4 column volumes (CV) of Heparin Buffer A to remove nonspecifically bound proteins.

Approximately 3 mg of protein eluted from Heparin column was loaded onto HiLoad 26/600 Superdex 200pg size-exclusion column (Cytiva) pre-equilibrated with buffer containing 10 mM HEPES, pH 7.5, 150 mM NaCl, and 0.5% Glycerol. Chromatography was performed at a flow rate of 2.4 mL/min. Pooled fractions from the Superdex column were diluted to adjust the final NaCl concentration to 50 mM and loaded onto a Bio-Rad ENrich Q10 х 100 anion exchange column equilibrated with Buffer A (10 mM HEPES, pH 7.5, 50 mM NaCl, and 0.5% Glycerol). After washing with 5 CV of Buffer A, bound proteins were eluted using a 20 CV linear gradient from 0% to 100% Buffer B (10 mM HEPES, pH 7.5, 1 M NaCl, 0.5% Glycerol). Eluted fractions containing the target protein were pooled, dialyzed against storage buffer (10 mM HEPES, pH 7.5, 150 mM NaCl, 0.5% Glycerol), and concentrated to 9 mg/mL.

RECQL5b, the largest spliced isoform of RECQL5 was expressed as a C-terminal fusion with the self-cleaving Mxe-CBD affinity tag in Escherichia coli Rosetta (DE3) pLysS cells (Novagen), as described (128). Protein expression was induced with 0.2 mM IPTG for 18 h at 16 °C. Cells were harvested by centrifugation and stored at –80°C. All the subsequent steps were carried out at 4 °C. Extract from 10 g of cell paste was prepared by sonication in 50 ml of cell breakage buffer (50 mM Tris-HCl, pH 7.5, 20% sucrose, 0.5 mM EDTA, 600 mM KCl, 0.01% Igepal, and the following protease inhibitors: aprotinin, chymostatin, leupeptin, and pepstatin A at 3 μg/ml each). The extract was clarified by ultracentrifugation (100,000 g for 90 min) and then mixed with 2 ml of chitin beads (New England Biolabs) for 2 h. The beads were washed with 60 ml of K buffer (20 mM KH_2_PO_4_, pH 7.4, 10% glycerol, 0.5 mM EDTA, 1 mM DTT, 0.01% Igepal, and protease inhibitors) containing 1 M KCl. Self-cleavage of the affinity tag was conducted in 0.5 ml of K buffer containing 100 mM KCl and 50 mM DTT for 18 h. The released RECQL5 protein was collected into 10 ml of K buffer with 100 mM KCl and fractionated in a 3-ml SP Sepharose column, which was washed with 30 ml of K buffer containing 150 mM KCl and then eluted with a 30-ml gradient of 150–600 mM KCl in K buffer. The peak fractions (~320 mM KCl) were pooled, diluted with an equal volume of K buffer, and loaded onto a 1-ml Source S column (GE Healthcare). The column was washed with 10 ml of K buffer containing 150 mM KCl and then eluted with a 12-ml gradient of 150–500 mM KCl in K buffer. Peak fractions (~300 mM KCl) were pooled and concentrated in a Centricon-30 device (Millipore) and stored in small aliquots at –80 °C. Approximately 2 mg of highly purified RECQL5 was obtained.

**Protein extraction and Western blot analysis**

Total proteins were extracted from cell lines using cell lysis buffer [50mM Tris, pH 7.5, 30 mM MgSO_4_, 8 mM EDTA, 2 mM dithiothreitol (DTT) and 2% Triton X-100)] supplemented with protease inhibitors. Cell lysates were clarified by centrifugation at 13,000 rpm for 15 min at 4^o^C. Protein concentrations were determined using the Bradford method (Cat.# 500-0205, Bio-Rad).

For WB analysis, 20-35µg of total protein was resolved by SDS-PAGE on 8–12% sodium dodecyl sulfate-polyacrylamide gels (SDS-PAGE) and transferred onto PVDF membranes (Cat.# IPVH00010, Fisher Scientific). Transferred membranes were blocked with 5% none-fat milk in PBS-T (0.05% Tween-20) for 1 h at RT and incubated with the indicated primary antibodies diluted in PBS-T for overnight (18 h) at 4^o^C. Membranes were washed three times for 10 min each with PBS-T and incubated with the appropriate HRP-conjugated secondary antibody for 1 h at RT, with gentle shaking. Following incubation, membranes were washed three additional times for 10 min each in PBS-T. Protein signals were detected using SuperSignal West Pico PLUS Chemiluminescent substrate (Cat.#PI34578, Fisher Scientific) and visualized using a chemiluminescence imaging system. Molecular weights markers were indicated on the left side of each blot. β-actin antibody used as a loading control. Detailed information on antibodies is provided in the reagents table.

**Co-immunoprecipitation (Co-IP) and chromatin isolation assays**

BRCA1-def. MDA-MB-436, PCNA^WT^ or PCNA^K164R^ RPE-1 cells seeded at a density of 5 × 10^6^ cells in 15-cm dishes.

For Co-IP data shown in Figure 4A, Figure 8C, Figure 8F, Figure S5K, Figure S6A–S6D, Figure S7C and Figure S7D, BRCA1-def MDA-MB-436 cells were treated with or without HU (5mM) for the indicated times (0, 1, or 2 h). Cells were harvested by scraping and collected by centrifugation at 13,000 rpm for 5 min at 4^o^C. Cell pellets were lysed in IP lysis buffer (Cat.#87788, Fisher Scientific) supplemented with protease inhibitors for 30 min on ice. Lysates were then centrifuged at 13,000 rpm for 15 min at 4^o^C, and the supernatants were collected. A total of 3% of the lysate was reserved as input, while the remaining lysate was subjected to immunoprecipitation using 2 μg of the indicated primary antibody overnight at 4^o^C with rotation. The following day, samples were incubated with 15 μL of dynabeads protein G (Cat.# 10004D, Thermo Scientific) for 1 h at RT with rotation. Beads were subsequently washed three times with IP lysis buffer for 10 min each at 4^o^C. Bound proteins were eluted with 4× SDS sample buffer and boiled at 100^o^C for 5 min. Samples were separated on 8%–12% SDS-PAGE gels, followed by WB analysis.

For Co-IP data shown Figure 7B, Figure 7E–7F, in Figure 4B, Figure 8D, Figure S6C, cells were transfected with 6.25 μg of the indicated DNA plasmids using FuGENE6 transfection reagent (Cat.#E2691, Promega) according to the manufacturer’s instructions and incubated for an additional 36 h. Cells were treated with HU (5mM, 1 h), where indicated, and subsequently subjected to the immunoprecipitation procedure described above. In selected experiments shown in Figure 4B, Figure 8D, and Figure S6C, cell lysates were additionally treated with DNase I (0, 25 U, or 50 U; Cat.#EN0521, Fisher Scientific) at 37^o^C for 30 min prior to immunoprecipitation.

For chromatin isolation assays, HEK293 cells were transfected with 6.25 μg empty vector (EV) or FLAG-RPRD1B plasmid for 48 h, whereas BRCA1-def MDA-MB-436 cells were treated with 30 nM siCtrl or RPRD1B siRNA for 36 h. Cells were then refreshed with complete growth DMEM medium containing or lacking 5 mM HU and incubated for 18 h. Subsequently, cells were re-incubated in complete growth DMEM medium for the indicated times (0, 1, or 2 h). Cells were harvested by scraping and collected by centrifugation at 13,000 rpm for 5 min at 4^o^C, and subjected to chromatin fractionation using the Chromatin Prep Module (Thermo Scientific Pierce^TM^, Cat.#26158), according to the manufacturer’s instructions.

Briefly, cells were washed twice by ice-cold PBS and cross-linked with 10 mL of 1% paraformaldehyde in PBS for 15 min with gentle rotation. Cross-linking was quenched with 1 × glycine solution for 5 min at RT with gentle rotation. The formaldehyde/glycine solution was aspirated, and cells were washed once with 10 mL of ice-cold PBS. Cells were scraped into 1 mL of ice-cold PBS containing 10 μL of the Halt Protease and Phosphatase Inhibitor Cocktail and transferred to a 1.5 mL microcentrifuge tube, followed by centrifugation at 3000 × g for 5 min at 4^o^C. Cell pellets were incubated in Lysis Buffer 1 (100 μL Membrane Extraction Buffer supplemented with 1 μL of the Halt Cocktail) for 10 min on ice and centrifuged at 9000 × g for 5 min to remove the supernatant. Pellets were then resuspended in 100 μL of MNase Digestion Buffer Working Solution supplemented with 0.25 μL of Micrococcal Nuclease (10 U/μL) and incubated in a 37^o^C water bath for 15 min with gentle inversion every 5 min. Subsequently, 20 μL of MNase Stop Solution was added to terminate the reaction, and samples were incubated on ice for 5 min, followed by centrifugation at 9000 × g for 5 min to recover nuclei. Nuclei were resuspended in 50 μL of Lysis Buffer 2 (50 μL of Nuclear Extraction Buffer supplemented with 0.5 μL of the Halt Protease and Phosphatase Inhibitor Cocktail) and incubated on ice for 15 min with vortexing for 15 s every 5 min. Samples were then centrifuged at 9,000 × g for 5 min, and the resulting supernatant containing the digested chromatin fraction was collected and subjected to WB analysis as described above.

**Immunofluorescence, nuclear structure assays and DNA damage foci**

For immunofluorescence (IF) staining, 2 × 10^5^ cells were seeded onto glass coverslips and transfected with 30 nM siCtrl or siRPRD1B. Forty-eight hours post-transfection, cells were washed twice with PBS (5 min each) and fixed in 4% paraformaldehyde (Cat.# 15710, Thermo Fisher Scientific) in PBS for 15 min at RT. Following fixation, cells were washed three times with PBS (5 min each) and permeabilized with 0.25% Triton X-100 (Cat.# T6767-100ML, Sigma-Aldrich) for 10 min. Cells were then washed three times (5 min each) and blocked with 3% BSA in PBS for 1 h at RT.

For chromosomal segregation analysis, cells were co-stained in the same samples with α-tubulin antibody (Cat# 11224-1-AP, Proteintech; 1:100 dilution) overnight at 4^o^C, together with phospho-MPM2 antibody (Cat.# 16-220, EMD Millipore/Fisher; directly conjugated, 1:100 dilution). After washing with PBS, cells were incubated with Alexa 488 Fluor-conjugated goatanti-rabbit IgG secondary antibody (Cat.# A11034, Thermo Fisher Scientific; 1:500 dilution) against α-tubulin (1:500 dilution) for 1 h at RT in the dark. Cell nuclei were counterstained with DAPI (Cat.# OB010020, Fisher Scientific). Fluorescence images were captured using a Zeiss LSM710 confocal microscope. Mitotic index was calculated as the percentage of mitotic cells relative to total DAPI-stained nuclei, with 100 cells analyzed per condition. Data are presented as mean ±S.D from three independent experiments.

For DNA damage foci analysis, cells were incubated with γ-H2AX antibody (Abcam, Cat.#ab11174, 1:100 dilution) overnight at 4^o^C. Cells were then washed with PBS and incubated with Alexa 488 Fluor–conjugated goat anti-mouse IgG secondary antibody (Thermo Fisher Scientific, Cat.#A11001; 1:500 dilution). Cell nuclei were counterstained with DAPI (Cat.# OB010020, Thermo Fisher Scientific). Fluorescence images were captured using a Zeiss LSM710 confocal microscope. γ-H2AX foci were quantified manually using ImageJ (NIH) by scoring individual nuclei identified by DAPI staining, and only intact, non-overlapping nuclei were included in the analysis. Cells containing ≥ 10 γ-H2AX foci were classified as γ-H2AX-positive unless otherwise specified. At least 100 nuclei per condition were analyzed per condition. Data shown are mean ±S.D from five independent experiments.

For nuclear structure and DNA damage foci analyses, cells were stained with DAPI. Fluorescence images were captured using Zeiss LSM710 confocal microscope to assess nuclear abnormalities, including micronuclei formation and nuclear bridging, as previously described.[3] Micronuclei were quantified as the percentage of cells containing one or more micronuclei relative to the total number of DAPI-stained cells. Approximately 100 nuclei per condition were analyzed per condition. Data shown are mean ±S.D from five independent experiments.

**Cell cycle arrest assay**

For cell cycle analysis, 2 × 10^5^ cells of BRCA1-def or rep- MDA-MB-436, HCC1937 or UWB1.289 cells, as well as BRCA1 wild type MCF7 cells in 6-well plates.

Cells were transfected with either 50 nM miRNA control or 50 nM miR-4485-3p or with 30 nM siCtrl or 30 nM siRPRD1B, as indicated. Three days after miRNA transfection, or two days after siRNA transfection, cells were harvested by trypsinization and washed twice with Ca^2+^/Mg^2+^-free PBS by centrifugation at 1000 × g for 5 min at 4^o^C.

Cells were then fixed by resuspension in 70% ethanol and incubated at 4^o^C for 2 h. Fixed cells were collected by centrifugation at 500 × g for 5 min at 4^o^C, and resuspended in 500 μL Propidium Iodide (PI)/Triton X-100 staining solution containing 0.1% (v/v) Triton X-100, RNase A (final 100 μg/mL), and PI (final 50μg/mL) in PBS. Cells were incubated for 30 min at RT in the dark, placed on ice, and analyzed by flow cytometry using a BD LSR II flow cytometer. Data are presented as mean ±S.D from triplicate experiments.

**I-SceI-based MMEJ (EJ2-GFP) and NHEJ (EJ5-GFP) reporter assays**

U2OS EJ2-GFP and U2OS EJ5-GFP cells containing integrated GFP-based DNA double-strand break (DSB) repair reporter constructs were used to measure microhomology-mediated end joining (MMEJ) and non-homologous end joining (NHEJ), respectively. Cells were trypsinized and seeded at a density of 3 × 10^5^ cells per well in 6-well plates to achieve approximately 60–70% confluency at the time of siRNA transfection.

Cells were first transfected with 30 nM siCtrl or siRPRD1B using Lipofectamine RNAiMAX (Thermo Fisher Scientific), according to the manufacturer’s instructions. Twenty-four hours after siRNA transfection, cells were transfected with 2.1 μg of either empty vector (EV) or HA-tagged I-SceI expression vector (pCBASceI, Cat.# 44024, Addgene) using jetPEI® transfection reagent (Polyplus-transfection; Genesee Scientific, Cat.# 55-102N) at a 1:2 DNA-to-jetPEI ratio in serum-free Opti-MEM medium, according to the manufacturer’s instructions, to induce site-specific DSBs.

Forty-eight hours after I-SceI transfection, cells were harvested by trypsinization and divided into two fractions. One fraction was subjected to WB analysis to confirm siRNA-mediated knockdown efficiency and protein expression levels. The remaining fraction was analyzed by flow cytometry using a BD LSR II flow cytometer. Cells transfected with EV vector were used to establish background fluorescence and to set gating thresholds for flow cytometry analysis. A total of 30,000 events were analyzed per sample, and DNA repair efficiency was calculated as the percentage of GFP-positive cells relative to the total cell population. All experiments were independently repeated three times.

**iPOND (isolation of proteins on nascent DNA) assay**

iPOND was performed as previously described with minor modifications. HEK293 cells were seeded at a density of 2× 10^7^ cells in 15-cm dish and cultured overnight to reach approximately 70–80% confluency at the time of transfection. The following day, cells were transiently transfected with 6.25 µg of empty vector (EV) (Cat.# CV011 from Sino Biological) or 6.25 µg of Myc-DDK-RPRD1B plasmid (Cat# RC207481 from OriGene Technologies, Inc.) and incubated for 36 h.

To label nascent DNA, cells were pulse-labeled with 10 µM EdU (Cat.# A10044, Thermo Fisher Scientific) for 30 min. Cells were then treated with 5 mM hydroxyurea (HU) for 1 or 2 h or left untreated to induce replication stress. At each time point, cells were crosslinked with 1% paraformaldehyde for 20 min at RT, quenched with 0.125M glycine (final concentration, 1/10 volume added), scraped, and collected by centrifugation at 4^o^C for 10 min, followed by three washes with ice-cold PBS.

EdU-labeled cells were permeabilized with 0.25% Triton X-100 in PBS for 30 min at RT in the dark. Cells were then washed twice by PBS, subsequently conjugated with 20 μM biotin-azide (Cat.# B10184, Thermo Fisher Scientific), 10 mM sodium L-ascorbate (Cat.# A4034-100G, Sigma-Aldrich), and 2 mM CuSO_4_ (Cat.# S0505-250G, Sigma-Aldrich) in 1 × PBS (10 mL per sample) for 2 h at RT in the dark. The no-click negative control was processed in parallel by omitting biotin-azide. After click chemistry, samples were washed twice with ice cold PBS, and lysed in buffer containing 50 mM Tris-HCl, pH 8, and 1% SDS supplemented with protease inhibitors (aprotinin and leupeptin). Chromatin was sonicated in cold water using a probe sonicator (30 s ON / 30 s OFF for 16 cycles at 8 mA) until lysates became translucent, followed by centrifugation at 16,000 × g for 10 min, and insoluble debris was removed by centrifugation. Supernatants were collected, and 5% was saved as input for WB analysis prior to streptavidin pull-down.

The remaining supernatant proteins bound to EdU-labeled DNA was diluted 1:1 with PBS containing protease inhibitors, and pulled down with Pierce^TM^ streptavidin agarose beads (Cat.# PI20347, Fisher Scientific) per sample overnight (~16–20 h) at 4^o^C. Streptavidin-agarose beads were washed once with lysis buffer (no additives added), once with 1 M NaCl for 5 min, and twice with lysis buffer (5 min each), with rotation during each wash step. Captured proteins were eluted by boiling in 2× SDS sample buffer at 95^o^C for 30 min and analyzed by WB.

**DNA fiber assays**

DNA fiber analysis was performed to assess replication fork stalling and restart in BRCA1-def MDA-MB-436 and HCC1937 cells, as well as BRCA1 wild-type MCF7 cells, following transfection with either 30 nM siCtrl or 30 nM siRPRD1B. Cells were seeded at a density of 2 × 10^5^ cells per dish in 6-cm dishes and cultured overnight prior to transfection.

Forty-eight hours post-transfection, cells were pulse-labeled with 25 µM 5-chloro-2’-deoxyuridine (CldU; Cat.# C6891-100MG, Sigma-Aldrich) for 30 min at 37^o^C in 5% CO_2_ using complete growth medium. DMEM was used for BRCA1-def MDA-MB-436 and BRCA1 wild-type MCF7 cells, whereas RPMI was used for BRCA1-def HCC1937 cells. Cells were incubated for 30 min at 37^o^C in 5% CO_2_. After incubation with CldU, cells were washed twice with fresh medium and treated with 5 mM HU for 2 h to induce replication fork stalling. Cells were then washed twice with fresh medium, and pulse-labeled with 125 µM 5-iodo-2’-deoxyuridine (IdU; Cat.#I7125-5G, Sigma-Aldrich) for 30 or 60 min at 37^o^C in 5% CO_2_.

After labeling, cells were trypsinized and collected by centrifugation. Cells were mixed with low-melting agarose, and agarose plugs were made. Agarose plugs were chilled at 4^o^C for 30 min to solidify the agarose. Each plug was mixed with 200 μL 0.5M EDTA, 25 μL sarkosyl, and 50 μL proteinase K and incubated at 50^o^C for 18 h. Plugs were washed three times with TE buffer containing 10 mM Tris (pH 8.0) and 1 mM EDTA at RT and placed in a reservoir containing 1 mL of MES buffer (pH 5.5). These reservoirs containing plugs were incubated at 65^o^C for 30 min to melt the agarose. The melted agarose was treated with 2 μL β-agarase (New England Biolabs) at 42^o^C for 14–18 h. After digestion with β-agarase, these reservoirs were stored at 4^o^C for 2–3 days before DNA fiber processing. DNA fibers were processed on slides (GenomicVision, Bagneux, FR) using the FiberComb molecular combing system. The newly synthesized CldU and IdU tracks were labeled (for 2.5 h in the dark, at RT) with antibodies recognizing CldU and IdU, followed by 1 h incubation with appropriate fluorophore-conjugated secondary antibodies (listed in the reagents table) at RT in the dark. Slides were mounted in PermaFluor aqueous self-sealing mounting medium (Thermo-Fisher Scientific, MA, USA), and images of DNA fibers were captured with a confocal Olympus FV1000D scanning microscope (Olympus America Inc., Center Valley, PA, USA). DNA fiber images were analyzed using ImageJ software, and at least 100 tracks were quantified for each experiment.

For fork degradation assay, BRCA1-def HCC1937 cells were seeded at a density of 2 × 10^5^ cells per dish in 6-cm dishes and cultured overnight. The following day, cells were transfected with either 30 nM siCtrl or 30 nM siRPRD1B. Forty-eight hours post-transfection, cells were pulse-labeled with 25 μM CldU for 30 min at 37^o^C, washed twice with fresh medium, and subsequently pulse-labeled with 125 μM IdU for 30 min at 37^o^C. Cells were washed twice with fresh medium and incubated with 5 mM HU for 0, 2, or 3 h. Cells were trypsinized and collected by centrifugation. Cells were processed for DNA fiber analysis as described above.

***In vitro* protein pulldown assays**

For Fig. 6C–D, 5 × 10^6^ BRCA1-def. MDA-MB-436 cells were seeded in 15-cm dishes and cultured overnight. The following day, cells were transfected with either 40 nM siRECQL5 or 30 nM siRPRD1B for 36 h. The culture medium was subsequently replaced with fresh DMEM, and cells were transiently transfected with either 6.25 µg of with either CMV control, FLAG-RECQL5 or HA-RPRD1B plasmids using FuGENE6 transfection reagent (Cat.#E2691, Promega) according to the manufacturer’s instructions and incubated for an additional 36 h.

Cells were then treated with or without 5 mM HU for 1 h prior to harvesting. Cell lysates were prepared using Pierce IP lysis buffer supplemented with protease inhibitors. Lysates were incubated o/n at 4oC on a rotating platform with either anti-HA or anti-FLAG magnetic beads. The following day, beads were washed twice with IP lysis buffer for 5 min each, and thrice with PBST for 5 min each at RT. HA-RPRD1B or FLAG-RECQL5 proteins were eluted using Elution Buffer A (0.15 M Glycine, pH 2.5) for 15 min at RT with rotation,, and immediately neutralized with Neutralization Buffer (1 M Tris-HCl, pH 8.0).

Purified HA-RPRD1B protein was subsequently incubated o/n at 4^o^C on a rotator with FLAG-RECQL5 protein complexes immobilized on FLAG magnetic beads. The following day, the complexes were washed thrice with IP lysis buffer and twice with PBST at RT with rotation. Bound proteins complexes were eluted using Elution Buffer A for 15 min at RT and immediately neutralized with Neutralization Buffer.

Eluted proteins were mixed with 4 ×SDS sample buffer and boiled at 95^o^C for 8 min. Protein samples were resolved by SDS-PAGE and transferred onto PVDF membranes using standard wet-transfer conditions. Membranes were blocked with 5% non-fat dry milk in PBST for 1 h at RT and incubated o/n at 4^o^C with either anti-HA antibody (Cat.#26183, ThermoFisher Scientific) or anti-FLAG antibody (Cat.#14-6681-80, Thermo Fisher Scientific). After washing, the membranes were incubated with HRP-conjugated secondary antibodies for 1 h at RT, and protein signals were detected using enhanced chemiluminescence (ECL) reagents.

For the Fig. S6A, 2 µg of His-tagged RPRD1B protein was incubated with 2 µg of untagged Ub-PCNA in a 15 µl reaction containing Tris buffer (25 mM Tris-HCl pH 7.5; 10% glycerol; 0.5 mM EDTA; 0.05% IGEPAL CA-630; 100 mM KCl; 5 mM β-mercaptoethanol) for 1 h at 4 °C with gentle agitation. The reaction mixture was then incubated with 5 µl of pre-equilibrated TALON beads for 30 min at 4 °C with agitation. Beads were washed twice with 100 µl of Tris buffer, and bound proteins were eluted with SDS-PAGE loading buffer. Eluted proteins were analyzed by SDS-PAGE followed by Coomassie staining, as indicated.

**Statistical Analyses**

All data are represented as mean ± standard deviation (SD) from at least three independent experiments, unless otherwise specified. Statistical analyses were performed as indicated in each figure legend. P values are shown in the corresponding figures, and statistical significance is denoted as follows: * = P ≤0.05; ** = P ≤0.01; *** = P ≤0.001. Unless otherwise specified, statistical significance was determined using two-sided unpaired Student’s t-test.

**Key Resource Table**

| **REAGENT or RESOURCE** | **SOURCE** | **IDENTIFIER** |
| --- | --- | --- |
| **Antibodies** |  |  |
| RPRD1B | Proteintech | Cat. #24542-1-AP; RRID: AB_2879597 |
| RPRD1B | Cell Signaling Technology | Cat.# 74693; RRID: N/A |
| RPRD1B | Sino Biological | Cat.# 14027-H07H; RRID: N/A |
| RPRD1A (P15RS) | Proteintech | Cat.# 23652-1-AP; RRID: AB_2879306 |
| pATR (S428) | Cell Signaling Technology | Cat.# 2853S; RRID: AB_2290281 |
| pATR (T1989) | Cell Signaling Technology | Cat.# 30632S; RRID: AB_2798992 |
| ATR | Cell Signaling Technology | Cat.# 2790S; RRID: AB_2227860 |
| pATM (S1981) | Cell Signaling Technology | Cat.# 5883P |
| ATM | Proteintech | Cat.# 27156-1-AP |
| pCtIP (T847) | Abcam | Cat.# ab254068; RRID: N/A |
| CtIP | Abcam | Cat.# ab70163; RRID: AB_1209429 |
| pRPA32 (Ser33) | Cell Signaling Technology | Cat.# 10148S; RRID: AB_3099645 |
| RPA32 | Bethyl Laboratories | Cat.# A300-244A; RRID: AB_185548 |
| p-EXO1 (T732) | Custom (Abmart) | Cat.# N/A; RRID: N/A |
| EXO1 | Proteintech | Cat.# 16253-1-AP; RRID: AB_2278140 |
| pChk1 (S317) | Cell Signaling Technology | Cat.# 8191S; RRID: AB_10859365 |
| pChk1 (S345) | Cell Signaling Technology | Cat.# 2348S; RRID: AB_331212 |
| Chk1 | Cell Signaling Technology | Cat.# 2360S; RRID: AB_2080320 |
| pChk2 (T68) | Cell Signaling Technology | Cat.# 2197T |
| tChk2 | Cell Signaling Technology | Cat.# 6334S |
| pCDK1 (Tyr15) | Cell Signaling Technology | Cat.# 4529S; RRID: AB_560953 |
| pCDK1 (Thr14) | Cell Signaling Technology | Cat.# 2543S; RRID: AB_823465 |
| CDK1 | Cell Signaling Technology | Cat.# 9116S; RRID: AB_2074795 |
| γ-H2AX | Abcam | Cat.# ab11174; RRID: AB_420030 |
| β-actin | Sigma-Aldrich | Cat.# A2228-2000UL; RRID: AB_2223340 |
| RPA1 | Cell Signaling Technology | Cat.# 2267S; RRID: AB_10642427 |
| ATRIP | Cell Signaling Technology | Cat.# 97687; RRID: AB_2303839 |
| 53BP1 | Novus Biologicals | Cat.# NB100-304; RRID: AB_3070458 |
| PARP1 | Cell Signaling Technology | Cat.# 9532S; RRID: AB_11187166 |
| POLQ | Cell Signaling Technology | Cat.# 48160S; RRID: N/A |
| POLQ | Thermo Fisher Scientific | Cat.# PA5-39885; RRID: AB_2556434 |
| BRCA1 | Santa Cruz Biotechnology, Inc. | Cat.# sc-6954 |
| RAD51 | Cell Signaling Technology | Cat.# 8875S; RRID: AB_11219092 |
| RAD52 | GeneTex | Cat.# GTX54722; RRID: AB_2887878 |
| DNA2 | Proteintech | Cat.# 18727-1-AP; RRID: AB_3069499 |
| PCNA | Abcam | Cat.# ab29; RRID: AB_303394 |
| PCNA | Proteintech | Cat.# 24036-1-AP; RRID: AB_2879414 |
| Ubiquityl-PCNA (Lys164) | Cell Signaling Technology | Cat.# 13439; RRID: AB_2798219 |
| K-63-linkage specific polyubiquitin (D7A11) | Cell Signaling Technology | Cat.# 12930; RRID: AB_2798064 |
| H3 | Cell Signaling Technology | Cat.# 9715S; RRID: AB_331563 |
| HA-tag | Cell Signaling Technology | Cat.# 3724S; RRID: AB_1549585 |
| HA-tag | Thermo Fisher Scientific | Cat.# 26183; RRID: AB_10978021 |
| FLAG-tag | Proteintech | Cat.# 20543-1-AP; RRID: AB_11232216 |
| FLAG-tag | Thermo Fisher Scientific | Cat.# 14-6681-80; RRID: AB_2637364 |
| Rabbit IgG | Cell Signaling Technology | Cat.# 2729S; RRID: AB_1031062 |
| Mouse IgG | Cell Signaling Technology | Cat.# 5415S; RRID: AB_10829607 |
| Rat anti-BrdU | Abcam | Cat.# ab220076 ; RRID: AB_851994 |
| Mouse anti-BrdU | BD Biosciences | Cat.# 347583; RRID: AB_1645614 |
| Goat α-mouse Alexa Fluor 488 | Thermo Fisher Scientific | Cat.# A11001; RRID: AB_2534069 |
| Goat α-rabbit Alexa Fluor 594 | Thermo Fisher Scientific | Cat.# A11034; RRID: AB_10561522 |
| **Chemicals, peptides, and recombination proteins** |  |  |
| Hydroxyurea (HU) | Sigma-Alrich | Cat.# H8627-5G |
| 5-Ethynyl-2’-deoxyuridine (EdU) | Thermo Fisher Scientific | Cat.# A10044 |
| 5-Iodo-2’-deoxyuridine (LdU) | Sigma-Aldrich | Cat.# I7125-5G |
| 5-Chloro-2′-deoxyuridine (CldU) | Sigma-Aldrich | Cat.# C6891-100MG |
| Proteinase K | Fisher Scientific | Cat#25-530-049 |
| Lipofectamine RNAiMAX | Thermo Fisher Scientific | Cat.#13778150 |
| Fugene^®^6 Transfection Reagent | Promega | Cat.#E2692 |
| Penicillin/ Streptomycin | Thermo Fisher Scientific | Cat.# 15140122 |
| Super Signal West Pico Plus | Thermo Fisher Scientific | Cat.# PI34578 |
| Super Signal West Femto | Thermo Fisher Scientific | Cat.# PI34095 |
| NUPAGE 4-12% Bis-Tris Gel (15 wells) | Thermo Fisher Scientific | Cat.# NP0323BOX |
| NUPAGE 4-12% Bis-Tris Gel (12 wells) | Thermo Fisher Scientific | Cat.# NP0322BOX |
| NUPAGE 4-12% Bis-Tris Gel (10 wells) | Thermo Fisher Scientific | Cat.# NP0321BOX |
| 40% Acrylamide/Bis Solution, 19:1 | Bio-Rad | Cat.# 1610144 |
| MES SDS Running buffer (20X) | Thermo Fisher Scientific | Cat.# NP0002 |
| NuPage Transfer buffer (20X) | Thermo Fisher Scientific | Cat.# NP0006-1 |
| LDS sample buffer (4X) | Thermo Fisher Scientific | Cat.# NP0008 |
| Pierce IP lysis buffer | Thermo Fisher Scientific | Cat.# 87788 |
| Cycloheximide | Santa Cruz Biotechnology | Cat.# sc-3508 |
| Bortezomib | Selleck Chemicals | Cat.# S1013 |
| Albumin Bovine Serum (BSA) | Sigma Aldrich | Cat.# A7030-10G |
| EMS Paraformaldehyde 16%, EM Grade | Thermo Fisher Scientific | Cat.# 15710 |
| DAPI fluoromount G | Thermo Fisher Scientific | Cat.# OB010020 |
| Biotin azide | Thermo Fisher Scientific | Cat.# B10184 |
| Formaldehyde 37% (wt/vol) solution | Sigma Aldrich | Cat.# F1635-500ML |
| Glycine, free base | Thermo Fisher Scientific | Cat.# G36050500.0 |
| 10× PBS | Thermo Fisher Scientific | Cat.# MT-46013CM |
| Triton X-100 | Sigma Aldrich | Cat.# T8787-100ML |
| Dimethyl sulfoxide (DMSO) | Thermo Fisher Scientific | Cat.# AAJ66650AD |
| Copper (II) sulfate | Thermo Fisher Scientific | Cat.# C489 |
| Sodium L-ascorbate | Sigma Aldrich | Cat.# A4034-100G |
| Sodium dodecyl sulfate (SDS) | Millipore Sigma | Cat.#151-21-3 |
| Aprotinin | Sigma Aldrich | Cat.#10236624001 |
| Leupeptin | Sigma Aldrich | Cat.#11017101001 |
| Pierce streptavidin agarose | Thermo Fisher Scientific | Cat.#PI20347 |
| Dynabeads protein G | Thermo Fisher Scientific | Cat.#10004D |
| Tris-EDTA, 1× Solution, pH 8.0 | Thermo Fisher Scientific | Cat.#BP24731 |
| β-agarase I | New England Biolabs | Cat.# M0392S |
| YOYO-1 Iodide (491/509), 1mM solution | Thermo Fisher Scientific | Cat.# Y3601 |
| RECQL5-IN-1 | MedChemExpress | Cat.# HY-145685 |
| BlockAid Blocking Solution | Thermo Fisher Scientific | Cat# B10710 |
| **Critical Commercial Assays** |  |  |
| DNase I Reaction Kit | New England Biolabs | Cat.# M0303L |
| Duo-Luciferase HS assay kit | GeneCopoeia | Cat.# LF004 |
| Chromatin isolation kit | Thermo Fisher Scientific | Cat.# 26158 |
| jetPEI^®^ tranfection kit | Genesee Scientific | Cat.# 55-102N |
| ZymoPUREII^TM^ Plasmid minipep kit | Genesee Scientific | Cat.# 11-553 |
| ZymoPUREII^TM^ Plasmid Maxipep kit | Genesee Scientific | Cat.# D4203-A |
| Cell Counting Kit-8 | Dojindo | Cat.# CK04-20 |
| **Experimental Models: Cell Lines** |  |  |
| BRCA1-def. MDA-MB-436 | ATCC | Cat.# HTB-130; RRID: CVCL_0623 |
| BRCA1-pro. MDA-MB-436 | Referred in this paper [4] | N/A |
| BRCA1-def. HCC-1937 | ATCC | Cat.# CTL-2336; RRID: CVCL_0290 |
| BRCA1-pro. HCC-1937 | Referred in this paper [5] | N/A |
| BRCA1-def. UWB1.289 | ATCC | Cat.# CRL-2945; RRID: CVCL_B079 |
| BRCA1-pro. UWB1.289 | ATCC | Cat.# CRL-2946; RRID: CVCL_B078 |
| BRCA1-wt MCF7 |  |  |
| EJ2-GFP U2OS | A gift from Dr. Jeremy Stark [6] |  |
| EJ5-GFP U2OS | A gift from Dr. Jeremy Stark [6] |  |
| HEK293 | ATCC | Cat.# CRL-1573; RRID: CVCL_0045 |
| 293T (*PCNA^WT^* and *PCNA^K164R^*) | A gift from Dr. George-Lucian Moldovan [7] |  |
| hTERT RPE1 [*PCNA^WT^*; *PCNA^K164R^* mutants (including *A1*, *B1* and *2B10*) and *Rab18^-/-^*] | A gift from Dr. Anja-Katrin Bielinsky [8] |  |
| **Bacterial and virus strains** |  |  |
| DH5 competent cells | New England Biolabs | Cat.# C3040I |
| Oligonucleotides |  |  |
| mirVana^TM^ miRNA mimic | Thermo Fisher Scientific | Cat.# 4464058 |
| hsa-miR-4485-3p mirVana^®^ | Thermo Fisher Scientific | Cat.# 4464066 |
| Control siRNA | OriGene | Cat.# SR30004 |
| RPRD1B siRNA | Horizon Discovery | Cat.# L-013787-01-0020 |
| RPRD1B siRNA | Thermo Fisher Scientific | Cat.# 4392420 |
| RPRD1A siRNA | Horizon Discovery | Cat.# L-007734-00-0005 |
| RPA1 siRNA | Horizon Discovery | Cat.# L-015749-01-0020 |
| PCNA siRNA | Thermo Fisher Scientific | Cat. # 4390825 |
| RECQL5 siRNA | Horizon Discovery | Cat. # L-019338-00-0010 |
| BRCA1 siRNA | Horizon Discovery | Cat.# L-003461-00-0020 |
| **Recombinant DNA** |  |  |
| pCMV parental plasmid | Sino Biological | Cat.#CV011 |
| FLAG-RPRD1B | Origene | Cat.#RC207481 |
| pCDASceI plasmid | Addgene | Cat.#26477 |
| Myc-RPRD1B plasmid constructs | A gift from Dr. Zhijie Chang [9] |  |
| HA-RPRD1B plasmid | Sino Biological | Cat.#HG14027-NY |
| pcDNA5-FRT/T0-FLAG-RECQL5 plasmid | Addgene | Cat.#222620 |
| HA-Ubiquitin plasmid | Addgene | Cat.#18712 |
| FLAG-RECQL5 plasmid constructs | A gift from Dr. Yinlun Liu [10] |  |
| **Software and algorithms** |  |  |
| ImageJ | Referred in this paper [11] | <https://imagej.nih.gov/ij/>; RRID: SRC_002285 |
| GraphPad Prism | GraphPad software | <https://www.graphpad.com/>; RRID: SCR_016137 |
| Microsoft Excel | Microsoft | [www.microsoft.com/en-gb/](http://www.microsoft.com/en-gb/); RRID: SRC_016137 |
| Zeiss Zen 3.11 | ZEISS | [https://www.zeiss.com/microscopy/us/products/software /zeiss-zen.html](https://www.zeiss.com/microscopy/us/products/software%20/zeiss-zen.html); RRID: SCR_021022 |
| **Others** |  |  |
| Zeiss LSM710 Confocal Microscope | ZEISS | LSM710 (Laser Scanning Microscope); RRID: SCR_018063 |
| Countess Automated Cell Counter | Invitrogen | [www.invitrogen.com/countess](http://www.invitrogen.com/countess)  Cat.# C10281; RRID: N/A |
| Countess Slides | Thermo Fisher Scientific | Cat.# C10312; RRID: SRC_021022 |
| DNA combing coverslips | PolyAn | Cat.# 10490804; RRID: SRC_021022 |
| LSR II Flow Cytometer | Becton Dickinson (BD) Biosciences | BD LSR II Flow Cytometer (Becton Dickinson, model LSR II); RRID: N/A |

**CONTACT FOR REAGENT AND RESOURCE SHARING**

Further information and requests for resources and reagents should be directed to and will be fulfilled by the Lead Contact, Dr. Robert A. Hromas at the UT Health Science Center at San Antonio ([hromas@uthscsa.edu](mailto:hromas@uthscsa.edu)).

**REFERENCES**

1.	Nacson J, Krais JJ, Bernhardy AJ *et al.* BRCA1 Mutation-Specific Responses to 53BP1 Loss-Induced Homologous Recombination and PARP Inhibitor Resistance. *Cell Rep* 2018;24: 3513–3527.e3517. <https://doi.org/10.1016/j.celrep.2018.08.086>

2.	Tassone P, Tagliaferri P, Perricelli A *et al.* BRCA1 expression modulates chemosensitivity of BRCA1-defective HCC1937 human breast cancer cells. *Br J Cancer* 2003;88: 1285–1291. <https://doi.org/10.1038/sj.bjc.6600859>

3.	Wu Y, Lee SH, Williamson EA *et al.* EEPD1 Rescues Stressed Replication Forks and Maintains Genome Stability by Promoting End Resection and Homologous Recombination Repair. *PLoS Genet* 2015;11: e1005675. <https://doi.org/10.1371/journal.pgen.1005675>

4.	Johnson N, Johnson SF, Yao W *et al.* Stabilization of mutant BRCA1 protein confers PARP inhibitor and platinum resistance. *Proc Natl Acad Sci U S A* 2013;110: 17041–17046. <https://doi.org/10.1073/pnas.1305170110>

5.	Tassone P, Tagliaferri P, Perricelli A *et al.* BRCA1 expression modulates chemosensitivity of BRCA1-defective HCC1937 human breast cancer cells. *British Journal of Cancer* 2003;88: 1285–1291. <https://doi.org/10.1038/sj.bjc.6600859>

6.	Howard SM, Yanez DA and Stark JM. DNA damage response factors from diverse pathways, including DNA crosslink repair, mediate alternative end joining. *PLoS Genet* 2015;11: e1004943. <https://doi.org/10.1371/journal.pgen.1004943>

7.	Thakar T, Leung W, Nicolae CM *et al.* Ubiquitinated-PCNA protects replication forks from DNA2-mediated degradation by regulating Okazaki fragment maturation and chromatin assembly. *Nat Commun* 2020;11: 2147. <https://doi.org/10.1038/s41467-020-16096-w>

8.	Leung W, Baxley RM, Traband E *et al.* FANCD2-dependent mitotic DNA synthesis relies on PCNA K164 ubiquitination. *Cell Reports* 2023;42. <https://doi.org/10.1016/j.celrep.2023.113523>

9.	Li M, Lin Y, Wang J *et al.* CREPT promotes LUAD progression by enhancing the CDK9 and RNAPII assembly to promote ERK-driven gene transcription. *Theranostics* 2025;15: 8337–8359. <https://doi.org/10.7150/thno.115572>

10.	Li M, Xu X, Chang CW *et al.* SUMO2 conjugation of PCNA facilitates chromatin remodeling to resolve transcription-replication conflicts. *Nat Commun* 2018;9: 2706. <https://doi.org/10.1038/s41467-018-05236-y>

11.	Schneider CA, Rasband WS and Eliceiri KW. NIH Image to ImageJ: 25 years of image analysis. *Nature Methods* 2012;9: 671–675. <https://doi.org/10.1038/nmeth.2089>
