# Supplementary material for: PCNA–RECQL5–RPRD1B recruit repair components to stressed replication forks to promote survival of BRCA1-deficient cancer cells
Source: Nucleic Acids Res. 2026 Aug 27;54(16):gkag831. doi: 10.1093/nar/gkag831 (PMC13519111; doi:10.1093/nar/gkag831)
Supplement: Supplementary file 1 [file gkag831_supplemental_files.zip › Supplementary Data Figures.docx]

# **SUPPLEMENTAL FIGURES**

**Supplementary Figure 1. Characterization of miR-4485-3p in BRCA1-def cells**

**(A)** IRE1 suppresses the expression of selected miRNAs in BRCA1-def cells. Global miRNA expression profiling in BRCA1-def MDA-MB-436 cells following IRE1 depletion. Cells were transfected with siRNA targeting IRE1, and global miRNA expression was analyzed using microarray technology as previously described. Loss of IRE1 resulted in the upregulation of several miRNAs, including miR-4485-3p.

**(B–C)** miR-4485-3p does not induce DNA damage in BRCA1-pro cells. Representative confocal images of γ-H2AX foci formation (*left*) in BRCA1-pro MDA-MB-436 cells (B) and HCC1937 cells (C) following transfection with miR-Ctrl or miR-4485-3p. Cells were stained with γ-H2AX (green) and DAPI (blue). Quantification of γ-H2AX foci is shown (*right*). At least 100 nuclei per condition are analyzed. Data shown as mean ± S.D were obtained from five independent experiments (n = 5). n.s., not significant.

**(D–E)** miR-4485-3p does not induce mitotic arrest in BRCA1-pro cells. Representative confocal images (*left*) showing α-tubulin (green), p-MPM2 (red), and DAPI (blue) stained in BRCA1-pro MDA-MB-436 (D) and HCC1937 (E) cells following transfection with 50 nM miR-Ctrl or 50 nM miR-4485-3p. Quantification of the fraction of mitotic cells is shown (*right*). At least 100 cells per condition were analyzed. Data represent mean ± S.D. (n = 5); n.s., not significant.

**(F)** Differences in cell cycle distribution, following transfection of miR-Ctrl or miR-4485-3p. BRCA1-def and -pro MDA-MB-436, HCC1937 and UWB1.289 cells as well as BRCA1 WT MCF7 cells were transfected with 50 nM miR-Ctrl or 50 nM miR-4485-3p for 3 days. Cells were then fixed, stained with propidium iodide (PI), and analyzed by flow cytometry. (i) 15,000 cells in G0/G1, S and G2/M of the cell cycle were sorted based on DNA contents after staining with PI. (ii) Bar graphs of cell distributions are shown (n = 3) as mean ± S.D, *P ≤0.05; **P ≤0.01; ***P ≤0.001; n.s., not significant. (iii) WCLs corresponding to the samples analyzed by flow cytometry were subjected to WB using RPRD1B and β-actin antibodies.

**Supplementary Figure 2. Validation of RPRD1B as a BRCA1-def-associated vulnerability.**

**(A)** miR-4485-3p does not repress RPRD1A protein expression. WB analysis (*bottom*) shows RPRD1A protein levels following transfection with miR-4485-3p in BRCA1-def and BRCA1-pro MDA-MB-436 and HCC1937 cells. Densitometric quantification of RPRD1A expression (*top*) is presented as mean ± S.D. from three independent experiments (n = 3).

**(B)** Overexpression of exogenous RPRD1B rescues cell survival in RPRD1B-depleted BRCA1-def MDA-MB-436, HCC1937, or UWB1.289 cells. Cells were transfected with 30 nM siCtrl, 30 nM siRPRD1B for 24 h. siRPRD1B-transfected cells were subsequently transfected with EV or HA-RPRD1B and cultured for additional 36 h. (Bi–ii) Representative images (i) and quantification (ii) of clonogenic survival assays are shown. Data are shown as mean ± S.D. and are representatives from three independent experiments (n = 3). (Biii) WCLs corresponding to those in panel (Bi) were subjected to WB analysis using HA and β-actin antibodies.

**(C)** Representative images (i) and quantification (ii) of clonogenic survival assays and (iii) WB analysis in BRCA1-def and BRCA1-pro MDA-MB-436, HCC1937, and UWB1.289 cells, following transfection with either siCtrl or siRPRD1B. A second independent siRNA sequence targeting RPRD1B was used in these experiments. n=3 independent experiments performed in triplicate, * P≤ 0.05, **P ≤ 0.01, ***P ≤0.001, n.s. = not significant.

**(D–F)** RPRD1B depletion increases *de novo* DNA damage in BRCA1-def cells. Representative confocal images of γ-H2AX foci formation following RPRD1B depletion in BRCA1-def MDA-MB-436 cells (D, *left*), HCC1937 cells (E, *left*), and UWB1.289 cells (F, *left*). Cells were stained with γ-H2AX (green) and DAPI (blue). Quantification of γ-H2AX foci is shown in the *right* panels for each cell line. At least 100 nuclei per condition were analyzed for each cell line. Data shown as mean ± S.D were obtained from five independent experiments (n = 5). **P ≤ 0.01; ***P ≤ 0.001.

**Supplementary Figure 3. RPRD1B depletion induces replication stress signaling preferentially in BRCA1-def cells**

**(A–C)** DNA damage is not increased following RPRD1B depletion in BRCA1-pro cells. Representative confocal images of γ-H2AX foci formation in BRCA1-pro MDA-MB-436 (A, *left*), HCC1937 (B, *left*), and UWB1.289 cells (C, *left*), following siCtrl- or siRPRD1B-transfection. Cells were stained with γ-H2AX (green) and DAPI (blue). Quantification of γ-H2AX foci per nucleus is shown in the *right* panels. At least 100 nuclei per condition were analyzed for each cell line. Data are presented as mean ± S.D. from five independent experiments (n = 5). n.s., not significant.

**(D–E)** RPRD1B depletion induces constitutive DNA damage signaling in BRCA1-def cells, but to a lesser extent in BRCA1-pro cells. (A) Immunoblot analysis of phosphorylated ATR (S428 and T1989), CtIP (T847), RPA32 (S4/S8), EXO1 (T732), CHK1 (S317 and S345), CDK1 (Tyr15 and Thr14), and γ-H2AX in BRCA1-def/pro MDA-MB-436, HCC1937, and UWB1.289 cells, following 30 nM siCtrl- or 30 nM siRPRD1B- transfection. β-actin was used as a loading control. (B) Densitometric quantification of the phosphorylation levels of the indicated proteins is presented as mean ± S.D. from three independent experiments (n = 3). Signal intensities were normalized to β-actin.

**(F-G)** RPRD1B depletion induces neither ATM/CHK2- nor 53BP1-mediated DNA damage signaling in BRCA1-def cells. (F) Immunoblot analysis of phosphorylated ATM (S1981), 53BP1 (Thr543) and CHK (T68) in BRCA1-def MDA-MB-436, HCC1937 and UWB1.289 cells, following the 30 nM siCtrl- or 30 nM siRPRD1B-transfection. β-actin was used as a loading control. (G) Densitometric quantification of the phosphorylation levels of the indicated proteins is presented as mean ± S.D. from three independent experiments (n = 3). Signal intensities were normalized to β-actin.

**(H-I)** RPRD1B depletion does not induce DNA damage signaling in BRCA1-wild type MCF7. (H) Immunoblot analysis of phosphorylated ATR (S428 and T1989), ATM (S1981), 53BP1 (Thr543), CtIP (T847), RPA32 (S4/S8), EXO1 (T732), CHK1 (S317 and S345), CHK2 (T68), CDK1 (Tyr15 and Thr14), and γ-H2AX in BRCA1-wild type MCF7 cells, following 30 nM siCtrl- or 30 nM siRPRD1B- transfection. β-actin was used as a loading control. (I) Densitometric quantification of the phosphorylation levels of the indicated proteins is presented as mean ± S.D. from three independent experiments (n = 3). Signal intensities were normalized to β-actin.

**(J)** Representative images (*top*) and quantification (*bottom*) of clonogenic survival assays in BRCA1- wild type MCF7 cells, following transfection with either 30 nM siCtrl or siRPRD1B. Data represent the mean ± S.D. from three independent experiments performed in triplicate, unless otherwise indicated. *P ≤ 0.05.

**Supplementary Figure 4. Recruitment of RPRD1B and replication fork repair factors to stressed replication forks.**

**(A–B)** RPRD1B is recruited to chromatin following replication stress in BRCA1 wild type HEK293 cells. HEK293 cells overexpressing HA-RPRD1B were treated with 10 mM HU for 18 h to induce replication stress. Following HU release, chromatin fractions were isolated at the indicated time points (0, 1, or 2 h) and analyzed by WB. Histone H3 was used as a loading control. **(A)** Representative immunoblots showing chromatin-bound HA-RPRD1B and histone H3. **(B)** Densitometric quantification of HA-RPRD1B signal intensity normalized to histone H3. Data are presented as mean ± S.D. from three independent experiments (n = 3).

**(C–D)** iPOND analysis of RPRD1B localization at replication forks in BRCA1-def MDA-MB-436 cells. Replicating DNA was labeled for 20 min, followed by treatment with 5 mM HU for the indicated times (0,1, or 2 h) to stressed replication forks. After isolation of proteins on newly synthesized DNA, WB detected RPRD1B (C), with γ-H2AX and PCNA as the markers of replication stress. Histone H3 served as a loading control. (D) Densitometric analysis of iPOND-captured RPRD1B signals shown as mean from three independent experiments.

**(E–F)** RPRD1B is recruited to stressed replication forks in BRCA1 WT HEK293 cells. (E) Immunoblots from iPOND analysis of HEK293 cells overexpressing FLAG-RPRD1B. Cells were labeled with 10 μM EdU for 20 min to mark nascent DNA, followed by treatment with 5 mM HU for the indicated times (0, 1, or 2 h) to induce replication fork stalling. γ-H2AX and PCNA were used as markers of replication stress, while histone H3 served as a loading control. (F) Densitometric quantification of FLAG-RPRD1B normalized to histone H3. Data are presented as mean ± S.D. from three independent experiments (n = 3).

**(G–H)** iPOND analysis of LIG3, XRCC1 recruitment to stressed replication forks in BRCA1-def MDA-MB-436 (G) and HCC1937 (H) cells. Cells were transfected with siCtrl or siRPRD1B and labeled with EdU to mark nascent DNA, followed by treatment with HU (5 mM) for the indicated times (0, 1, or 2 h) to induce replication fork stalling. LIG3, XRCC1, RPRD1B and Histone H3 proteins associated with newly synthesized DNA were isolated and analyzed by WB. Cells were labeled with 10 μM EdU for 20 min to mark nascent DNA, followed by treatment with 5 mM HU for the indicated times (0, 1, or 2 h) to induce replication fork stalling. Histone H3 served as a loading control.

**(I)** RPA1 depletion reduces recruitment of multiple repair factors, including RPRD1B to stressed replication forks in BRCA1-def cells. BRCA1-def MDA-MB-436 cells were transfected with 40 nM siCtrl or 40 nM siRPA1 for 2 days, and subsequently exposed to 5 mM HU for the indicated times (0, 1, or 2 h). iPOND analysis was performed to assess recruitment of RPA1, ATR, 53BP1, PARP1, POLQ, RAD51, RAD52, and RPRD1B to replication forks. γ-H2AX and PCNA served as markers of replication stress response. Histone H3 was used as a loading control.

**(J)** iPOND analysis of RPRD1B localization at replication forks in BRCA1-wild type MCF7 cells. Cells were transfected with either (30 nM) siCtrl or (30nM) siRPRD1B for 2 days and subsequently subjected to iPOND assay. Replicating DNA was pulse-labeled for 20 min, followed by treatment with 5 mM HU for the indicated times (0, 1, or 2 h) to induce replication fork stalling. Proteins associated with newly synthesized DNA were isolated and analyzed by WB for RPA1, ATR, 53BP1, PARP1, POLQ, RPRD1B and Histone H3. Histone H3 served as a loading control.

**Supplementary Figure 5. Characterization of RPRD1B-associated replication fork repair complexes during replication stress.**

**(A)** RPRD1B associates with replication fork repair components upon replication stress in BRCA1-def cells. BRCA1-def MDA-MB-436 cells treated with 5 mM HU for the indicated times (0, 1, or 2 h) were subjected to Co-IP analysis. Antibodies against RPA1, ATR, ATRIP, POLQ, PARP1, 53BP1, RAD51, DNA Ligase III (LIG3), or control IgG were used to pull down proteins. WB analyses were then performed to detect specific proteins using the corresponding primary antibodies to detect specific proteins.

**(B)** RPRD1A associates only with RPA1 following replication stress. Cell lysates were prepared from BRCA1-def MDA-MB-436 cells treated with 5 mM HU for the indicated times (0, 1, 2 h) and subjected to Co-IP using antibodies against RPA1, 53BP1, POLQ, or control IgG. Immunoprecipitates were analyzed by WB with the indicated antibodies.

**(C)** Reverse Co-IP analysis confirms the interaction between RPA1 and RPRD1A.

**(D)** RPRD1A co-immunoprecipitation with RPA1 is DNA-dependent. Cell lysates were prepared from BRCA1-def MDA-MB-436 cells treated with 5 mM HU for 1 h. Lysates were then treated with DNase I (0 U, 25 U, or 50 U) for 30 min at 37^o^C prior to Co-IP. Immunoprecipitates were analyzed by WB using the indicated antibodies.

**(E)** The interaction between RPRD1A and RPA1 requires RPRD1B. BRCA1-def MDA-MB-436 cells were transfected with either 30 nM siCtrl or 30 nM siRPRD1B for 2 days, and subsequently treated with 5 mM HU for 1 h. Co-IP was performed using lysates from HU-treated cells following depletion of RPRD1B. Immunoprecipitates were analyzed by WB with the indicated antibodies.

**Supplementary Figure 6. RPRD1B is dispensable for c-NHEJ and does not alter mitotic progression or cell cycle distribution in BRCA1-pro and BRCA1 wild type cells**

**(A)** RPRD1B is unessential for canonical non-homologous end joining (c-NHEJ). U2OS EJ5 reporter cells were transfected with either siCtrl or siRPRD1B. After 24 h, cells were further transfected with either empty vector (EV) or HA-I-SceI plasmid. (i) Representative images of flow histograms of the EJ5 cells with and without I-SceI transduction. (ii) The knockdown efficiency of RPRD1B and the expression of HA-I-SceI were confirmed by WB analysis. (iii) Quantification of GFP-positive cells, indicating productive c-NHEJ repair, as measured by flow cytometry. Approximately 30,000 cells were analyzed per condition. Data are presented as mean ± S.D. from three independent experiments (n = 3). n.s., not significant.

**(B–D)** Mitotic arrest is not increased following RPRD1B depletion in BRCA1-pro cells. Representative confocal images (*left*) showing α-tubulin (green), MPM-2 (red), and DAPI (blue) staining in BRCA1-pro MDA-MB-436 (B, *left*), HCC1937 (C, *left*), and UWB1.289 cells (D, *left*) following transfection with 30 nM siCtrl or 30 nM siRPRD1B. At least 100 cells per condition were analyzed. Quantification of the percentage of mitotic cells is shown in the right panels for each cell line. Data are presented as mean ± S.D. from five independent experiments (n = 5). n.s., not significant.

**(E)** Differences in cell cycle distribution, following transfection of siCtrl or siRPRD1B. BRCA1-def and -rep MDA-MB-436, HCC1937 and UWB1.289 cells, and BRCA1 wild type MCF7 cells were transfected with 30 nM siCtrl or 30 nM siRPRD1B for 2 days. Cells were then fixed, stained with propidium iodide (PI), and analyzed by flow cytometry. (i) 15,000 cells in G0/G1, S and G2/M of the cell cycle were sorted based on DNA contents after staining with PI. (ii) Bar graphs of cell distributions are shown (n = 3) as mean ± S.D, *P ≤0.05; **P ≤0.01; ***P ≤0.001; n.s., not significant. (iii) WCLs corresponding to the samples analyzed by flow cytometry were subjected to WB using RPRD1B and β-actin antibodies.

**Supplementary Figure 7. Characterization of the interaction between RPRD1B, RECQL5, and CPNA**

**(A)** Purified RECQL5 interacts with purified RPRD1B *in* *vitro*. *In* *vitro* Talon pull-down assay was performed to assess the interaction between RPRD1B and RECQL5. S, supernatant; E, eluant; bead-bound fraction, proteins bound to Talon beads. His-tagged RPRD1B is detected in the RECQL5 pull-down eluant but not in the control Talon beads-only eluant.

**(B)** Polyubiquitinated PCNA does not directly interact with RPRD1B in vitro. *In vitro* Talon pull-down assay was performed to assess the interaction between RPRD1B and PCNA or Ub-PCNA. S, supernatant; E, eluant; bead-bound fraction, proteins bound to Talon beads. Ub-PCNA is not detected in the RPRD1B pull-down eluant.

**(C)** RPRD1B preferentially co-immunoprecipitates with WT PCNA but not with PCNAK164R. Cell lysates from RPE-1 cells stably expressing PCNA*^WT^*, PCNAK*^164R^* (clone A1), PCNAK*^164R^* (clone B1), and PCNAK164R (clone 2B10) were subjected to co-immunoprecipitation using anti-RPRD1B antibodies. Western blot analysis was performed with the indicated antibodies (left). Densitometric quantification of immunoprecipitated PCNA is shown (right). Data are presented as mean ± S.D. from three independent experiments (n = 3). ***, P ≤ 0.001.

**(D)** RAD18 depletion reduces the co-immunoprecipitation of RPRD1B with PCNA. Cell lysates from RPE-1 cells stably expressing PCNA*^WT^*, PCNAK*^164R^*, or PCNA*^Rad18−/−^* were subjected to co-immunoprecipitation using anti-PCNA antibodies, followed by western blot analysis to detect RPRD1B.

**Supplementary Figure 8. Characterization of replication fork dynamics following RPRD1B depletion using DNA fiber assays.**

A. BRCA1-def HCC1937 cells were transfected with 30 nM siCtrl or siRPRD1B. Cells were pulse-labeled with CldU for 30 min (red), followed by treatment with 5 mM HU for 120 min to induce replication stress, and subsequently pulse-labeled with IdU (green) for either 30 min or 60 min.

(i) Experimental scheme and representative images of DNA fiber assay. (ii) Analysis of stalled, restarted, and newly initiated replication forks by DNA fiber assay. (iii) Distribution of DNA fiber length ratios (IdU/CldU) grouped by ratio category. (iv) WB analysis showing RPRD1B protein depletion in the corresponding samples shown in panels (i–iii).

B. BRCA1-WT MCF7 cells were transfected with 30 nM siCtrl or siRPRD1B and subjected to the same CldU-HU-IdU labeling protocol as in (A).

(i) Experimental scheme and representative images of DNA fiber assay. (ii) Analysis of stalled, restarted, and newly initiated replication forks by DNA fiber assay. (iii) WB analysis showing RPRD1B protein depletion in the corresponding samples shown in panels (i–ii).

C. BRCA1-def HCC1937 cells were transfected with 30 nM siCtrl or siRPRD1B and sequentially pulse-labeled with CldU for 30 min (red) and IdU (green) for 30 min.

(i) Experimental scheme and representative images of DNA fiber assay. (ii) Analysis of stalled, restarted, and newly initiated replication forks by DNA fiber assay. (iii) WB analysis showing RPRD1B protein depletion in the corresponding samples shown in panels (i–ii).

D. BRCA1-def HCC1937 cells were transfected with 30 nM siCtrl or siRPRD1B, pulse-labeled with CldU for 30 min (red) and IdU (green) for 30 min, respectively, and then exposed to 5 mM HU in the indicated time course (0, 2 or 3 h).

(i) Experimental scheme and representative images of DNA fiber assay. (ii) Violin plot analysis of DNA fiber length ratios (IdU/CldU). (iii) WB analysis showing RPRD1B protein depletion in the corresponding samples shown in panels (i–ii).

**REFERENCES**

1.	Nacson J, Krais JJ, Bernhardy AJ *et al.* BRCA1 Mutation-Specific Responses to 53BP1 Loss-Induced Homologous Recombination and PARP Inhibitor Resistance. *Cell Rep* 2018;24: 3513–3527.e3517. <https://doi.org/10.1016/j.celrep.2018.08.086>

2.	Tassone P, Tagliaferri P, Perricelli A *et al.* BRCA1 expression modulates chemosensitivity of BRCA1-defective HCC1937 human breast cancer cells. *Br J Cancer* 2003;88: 1285–1291. <https://doi.org/10.1038/sj.bjc.6600859>

3.	Wu Y, Lee SH, Williamson EA *et al.* EEPD1 Rescues Stressed Replication Forks and Maintains Genome Stability by Promoting End Resection and Homologous Recombination Repair. *PLoS Genet* 2015;11: e1005675. <https://doi.org/10.1371/journal.pgen.1005675>

4.	Johnson N, Johnson SF, Yao W *et al.* Stabilization of mutant BRCA1 protein confers PARP inhibitor and platinum resistance. *Proc Natl Acad Sci U S A* 2013;110: 17041–17046. <https://doi.org/10.1073/pnas.1305170110>

5.	Tassone P, Tagliaferri P, Perricelli A *et al.* BRCA1 expression modulates chemosensitivity of BRCA1-defective HCC1937 human breast cancer cells. *British Journal of Cancer* 2003;88: 1285–1291. <https://doi.org/10.1038/sj.bjc.6600859>

6.	Howard SM, Yanez DA and Stark JM. DNA damage response factors from diverse pathways, including DNA crosslink repair, mediate alternative end joining. *PLoS Genet* 2015;11: e1004943. <https://doi.org/10.1371/journal.pgen.1004943>

7.	Thakar T, Leung W, Nicolae CM *et al.* Ubiquitinated-PCNA protects replication forks from DNA2-mediated degradation by regulating Okazaki fragment maturation and chromatin assembly. *Nat Commun* 2020;11: 2147. <https://doi.org/10.1038/s41467-020-16096-w>

8.	Leung W, Baxley RM, Traband E *et al.* FANCD2-dependent mitotic DNA synthesis relies on PCNA K164 ubiquitination. *Cell Reports* 2023;42. <https://doi.org/10.1016/j.celrep.2023.113523>

9.	Li M, Lin Y, Wang J *et al.* CREPT promotes LUAD progression by enhancing the CDK9 and RNAPII assembly to promote ERK-driven gene transcription. *Theranostics* 2025;15: 8337–8359. <https://doi.org/10.7150/thno.115572>

10.	Li M, Xu X, Chang CW *et al.* SUMO2 conjugation of PCNA facilitates chromatin remodeling to resolve transcription-replication conflicts. *Nat Commun* 2018;9: 2706. <https://doi.org/10.1038/s41467-018-05236-y>

11.	Schneider CA, Rasband WS and Eliceiri KW. NIH Image to ImageJ: 25 years of image analysis. *Nature Methods* 2012;9: 671–675. <https://doi.org/10.1038/nmeth.2089>
